# Silver Nanoparticles (AgNPs) from *Lysinibacillus* sp. Culture Broths: Antibacterial Activity, Mechanism Insights, and Synergy with Classical Antibiotics

**DOI:** 10.3390/biom15050731

**Published:** 2025-05-16

**Authors:** Carlos Pernas-Pleite, Amparo M. Conejo-Martínez, Irma Marín, José P. Abad

**Affiliations:** Department of Molecular Biology, Biology Building, Faculty of Sciences, Autonomous University of Madrid, Cantoblanco, 28049 Madrid, Spain; carlos.pernas@uam.es (C.P.-P.); amparoconejo96@gmail.com (A.M.C.-M.)

**Keywords:** AgNPs, antibacterial activity, antibiotic resistance, green synthesis, FICI, *Lysinibacillus* sp., ROS, silver nanoparticles, synergy

## Abstract

Antibiotic-resistant bacteria pose problems for infection prevention and treatment, so developing new procedures or substances against infection is mandatory. Silver nanomaterials are among the more promising antibacterial agents. Herein, we describe the biogenic synthesis of silver nanoparticles (AgNPs) using culture broths from an undescribed species of *Lysinibacillus*. Culture broths with or without NaCl and from the exponential and stationary growth phases produced four AgNP types. Nanoparticles’ shapes were quasi-spherical, with core sizes of 7.5–14.7 nm and hydrodynamic diameters of 48.5–80.2 nm. All the AgNPs contained Ag^0^ crystals and some AgCl ones. Moreover, their coronas presented different proportions of carbohydrates, proteins, and aliphatic compounds. The AgNPs were good antibacterial agents against six bacterial species, three Gram-positive and three Gram-negative, with MICs of 0.3–9.0 µg/mL. Their activity was higher against the Gram-negative bacteria and particularly against *Pseudomonas aeruginosa*. These AgNPs acted synergistically with several of the fifteen tested antibiotics. Interestingly, AgNP combinations with some of these inhibited the growth of antibiotic-resistant bacteria, as in the case of *S. epidermidis* for streptomycin and *S. aureus* for colistin. The ROS production by *E. coli* and *S. aureus* when treated with most AgNPs suggested different mechanisms for bacterial killing depending on the AgNP.

## 1. Introduction

Antibiotic resistance is one of the main problems that global health faces, with an estimated 39 million people expected to die from antibiotic-resistant infections between 2024 and 2050 [1]. Due to this, it is vital to search for new antimicrobials and restore the use of the classical ones, either by reversing the resistance or improving their effect by combining different antimicrobials.

Various alternative therapies have been experimented with to search for new antimicrobials, one of the most promising being the use of nanomaterials, including metal nanoparticles [2]. Due to the unique structural characteristics of these materials, such as their small size and high surface-to-volume ratio, their use for various applications has risen recently [2]. Among the different nanoparticles, the ones that have shown the better antimicrobial effect are silver nanoparticles (AgNPs) [3,4,5] due to their high efficacy in vitro and in vivo [6,7,8,9], against bacterial [6,10,11], fungal [12,13], and even viral infections [14]. The antimicrobial efficacy of these nanoparticles is related to their physicochemical characteristics, such as size, shape, Z-potential, or coating [3,15]. Therefore, the determination of the characteristics of these nanomaterials using various analytical techniques [11,16,17] is of great importance to establish relationships with their functionality.

The synthesis of AgNPs can use chemical, physical, or biological methods. In the biological ones, organic compounds of biological origin, extracts or culture media are used [10,18,19]. These methods have advantages over the chemical or physical ones as the use of methodologically simple protocols that do not require large energy expenditures or highly specialised machinery and that do not use toxic or environmentally hazardous substances [20], which is why they are “green” or “eco-friendly” syntheses [21]. In addition, the biological AgNPs synthesis can take advantage of the natural biodiversity to obtain different AgNP types with diverse characteristics when using different plants [22,23], algae [24], fungi [25], animal tissues [26,27] and simpler from bacterial cultures [10,28], the latter being either intracellular, or extracellular from culture broths [29]. The culture conditions are also of great importance for the characteristics of the obtained AgNPs since the composition of the medium [30,31,32], the temperature [33], and the pH of the culture medium [34] can affect the characteristics of the AgNPs and therefore their activity.

The AgNPs’ efficacy seems closely related to their ability to release Ag^+^ ions [35,36], like the AgNO_3_ used in treating certain skin infections [37]. Theoretically, these Ag^+^ ions can bind to different organic compounds, affecting several cellular mechanisms and ultimately inducing cell death [38]. The mechanisms of action of both AgNO_3_ and AgNPs most frequently mentioned in the literature are the production of reactive oxygen species (ROS) [39] and the disruption of envelope structures [40]. However, some authors consider the interference with protein synthesis or interaction with DNA [36], among others [41], so there is no clear idea of the mechanism of action of these nanomaterials.

One of the most efficient and popular ways to exploit the antimicrobial capabilities of AgNPs in recent years is their use in combination with classical antibiotics to achieve more efficient antimicrobial effects [42]. This synergistic effect of AgNPs with classical antibiotics has been studied for different types of silver nanoparticles using several techniques. The most frequently used ones measure the increase in the inhibition halo on a semi-solid medium or the variation of the minimum inhibitory concentration (MIC) in liquid media, as can be deduced from recent reviews on this subject [10,42]. However, another method, known as the chequerboard method, performed in a liquid medium and analysed through the calculation of the fractional inhibitory concentration index (FICI), is more suitable for this purpose [43,44,45], allowing us to determine positive synergy, independent activity, or antagonism based on a FICI scale, based on statistical considerations [44,45]. Making comparisons among the synergy results from different studies is very difficult due to the various analytical parameters used by their authors, so some researchers consider that an agreement on standards is urgent in this growing field.

Different bacterial species of the genus *Lisynibacillus* have been employed for different biotechnological uses, such as the degradation of pesticides like malathion [46], the synthesis of methanol with *L. xylanilyticus* [47], control of insects such as mosquitoes acting as disease vectors with *L. thuringiensis* and *L. sphaericus* [48], or production of silver nanoparticles [28,49,50].

The synthesis of AgNPs from bacteria of the genus *Lysinibacillus* has been described by several authors; for example, Bhatia et al. produced AgNPs with antimicrobial activity using broths of *L. boronitolerans* and *L. varians* cultured in basal salt medium [51,52], Huq et al. synthesised AgNPs from broths of *L. xylanilyticus* grown in R2A medium [50], Omole et al. obtained AgNPs using the cell extract of *L. fusiformis* [49], El-Bendary et al. [53] produced AgNPs of varying morphologies from extracellular broths of different strains of *L. sphaericus*, Narayanan et al. produced Ag/ZnO nanocomposites from *L. sphaericus* cultures grown in tryptic soy broth [28], and Liu et al. obtained AgNPs using proteins of *L. sphaericus* [54].

The objectives of this study were to extracellularly produce new types of AgNPs with culture supernatants of a species of the *Lysinibacillus* genus not previously used for this goal and to determine their physicochemical characteristics using different methodologies and their antimicrobial properties on their own using microdilution assays in liquid medium or combined with fifteen antibiotics using the chequerboard method, ending with assays to study the importance of ROS generation in the antimicrobial action of the AgNPs.

## 2. Materials and Methods

The protocols used in this work were based on previous work carried out by our research group, which facilitated the comparison of these results with those previously published [55,56]. Therefore, the following is a brief description of the techniques and methodologies, highlighting the differences or new contributions with respect to previous work.

### 2.1. Microorganisms, Culture Media Used, and Cell-Free Broth Preparation

The bacterium used for the AgNPs’ syntheses was previously isolated from the Tinto River estuary (Huelva) [57,58] and identified as isolate B140 UAM. Its phylogenetic assignment was performed based on the sequence of its 16S rDNA.

Bacterial genomic DNA extraction was performed from colonies on nutrient agar plate cultures using the Ultraclean Microbial DNA Isolation Kit (MOBIO Laboratories Inc., Carlsbad, CA, USA). The 16S rDNA was PCR amplified from the obtained genomic DNA (primers 27F and 1492R) using the amplification protocols and conditions described in previous work [56]. Macrogen (Amsterdam, The Netherlands) sequenced the amplified DNA, and the sequence (acc. No. PV186713) was aligned with those from the closest type species to generate the trees using the same tools employed in previous studies [55,56].

Bacterial strains used for antibacterial activity testing were the Gram-negative bacteria *Escherichia coli* ATCC 25922; *Klebsiella pneumoniae* ATCC 2966; and *Pseudomonas aeruginosa* CECT 108, PA01, and PA14; as well as Gram-positive species *Bacillus subtilis* 168; *Staphylococcus aureus* CECT 794; and *Staphylococcus epidermidis* ATCC 12228, cultured in nutritive liquid medium at 37 °C, as we described in previous works [55,56].

The *Lysinibacillus* sp. was cultured in 400 mL of nutritive medium with and without NaCl at 30 °C with shaking at 150 rpm. A total of 200 mL of the cultures was harvested on the exponential (18 h; A_660_ ≈ 0.45) and stationary phases (42 h; A_660_ ≈ 0.85). Finally, the cells and the broths were separated via centrifugation and filtration, keeping the broths at −20 °C following the same protocols previously described [56].

### 2.2. Green Synthesis of AgNPs

The AgNPs were synthesised under light at 22 °C, after adding to the broths extra pure AgNO_3_ (Merck, Darmstadt, Germany) at a 1 mM final concentration. Synthesis kinetics were followed by UV-Vis spectrophotometry until the absorbance reached its highest value. Thereafter, AgNPs were washed and concentrated following the same protocol for the AgNPs obtained from *Pseudomonas alloputida* in a previous study [56]; in brief, the AgNPs were sedimented via centrifugation, and the pellet was resuspended in Milli-Q water, repeating the procedure three more times, after which the pellet was resuspended in one-tenth of the original reaction volume in Milli-Q water and kept at 4 °C until used. The obtained AgNPs were named according to the presence or absence of NaCl in the broth used for their synthesis and the growth phase from which broths were sampled, as indicated in Table 1.

### 2.3. Determination of the AgNPs’ Physicochemical Properties

The AgNPs’ physicochemical properties assessment was performed as in previous works in our laboratory [55,56], using the same facilities. FLUOStar^®^ Omega (BMG, Labtech, Offenburg, Germany) equipment allowed us to perform the spectrophotometric analysis of the AgNPs. Size and shape were analysed with transmission electron microscopy (TEM) using a JEM1400 JEOL microscope (Tokyo, Japan), and the images were processed with ImageJ64 software (https://imagej.nih.gov/ij/download.html (accessed on 1 February 2023)) to determine AgNPs’ core sizes. Dynamic light scattering (DLS) and electrophoretic light scattering (ELS) were used to determine the hydrodynamic sizes and the Z-potentials, respectively, in a Zetasizer Ultra (Malvern Panalytical, Malvern, UK). Elemental composition and Ag concentration in AgNPs’ suspensions were analysed using total X-ray reflection fluorescence (TXRF). In this report, AgNPs’ concentrations were expressed as the Ag concentration. The AgNPs’ crystallinity was evaluated by X-ray diffraction (XRD). The organic compositions of the broths used in the AgNPs’ syntheses, of the broths remaining after the AgNPs’ purification, and that of the AgNPs coronas were determined using Fourier transform infrared spectroscopy (FTIR) analysis. The TXRF, XRD, and FTIR analyses were performed at the Servicio Interdepartamental de Investigación (SIdI) of the Universidad Autónoma de Madrid (UAM). DLS and ELS studies were performed using the UAM Material Physics Department’s equipment.

### 2.4. Evaluation of AgNPs’ Antibacterial Activity

The AgNPs’ antibacterial activities were assessed in triplicate via microdilution assays in 96-well plates. AgNP half serial dilutions were used against the control bacteria mentioned in Section 2.1 at 1.5 × 10^5^ CFUs/mL. The incubation was carried out overnight at 37 °C, with hourly absorbance measurements at 660 nm. The evaluated antibacterial activity parameters were the minimum inhibitory concentration (MIC), the 50% inhibitory concentration (IC_50_), the minimum bactericidal concentration (MBC) obtained from colony counting in nutritive agar medium, and the 50% inhibitory concentration of biofilm formation (ICb_50_) obtained using the crystal violet staining protocol [59]. With comparative goals, the same antibacterial parameters were obtained for AgNO_3_ and streptomycin (Sm). These parameters were expressed for all the silver-containing materials as their concentration of silver.

The antibacterial activity of the AgNPs conserved in the dark at 4 °C for one month and two years was evaluated after these times in the same conditions indicated above, using *E. coli* ATCC 25922 as the control bacterium.

### 2.5. Testing of the Putative Antibacterial Synergy of AgNPs with Classical Antibiotics

The synergistic effect of AgNPs with different classical antibiotics was studied using the chequerboard method [44,45], following the same protocols used in our previous studies [55,56]. The fractional inhibitory concentration index (FICI) [44] and the modulation factor (MF) of the antibiotic [60] (Figure S1) were calculated and evaluated using the following scale: FICI ≤ 0.5 and MF ≥ 4 positive synergy, FICI > 4.0 and MF < 0.5 antagonism. Each AgNP and AgNO_3_ was evaluated in combination with each of the chosen antibiotics: ampicillin (Ap), penicillin-G (Pn), rifampicin (Rp), streptomycin (Sm), and vancomycin (Vm) (Duchefa Biochemie, Haarlem, The Netherlands); ceftazidime (Cz), colistin (Co), ciprofloxacin (Cp), kanamycin (Km), nalidixic acid (Nx), and tetracycline (Tc) (Sigma-Aldrich Co., St. Louis, MO, USA); ertapenem (Ep) (Merck KGaA, Darmstadt, Germany); chloramphenicol (Cc) and erythromycin (Em) (Boehringer, Mannheim, Germany), and tigecycline (Tg) (Biosynth, Compton, UK).

### 2.6. Reactive Oxygen Species (ROS) Production and Bactericidal Activity of the AgNPs

The accumulation of the reactive oxygen species (ROS) by *E. coli* ATCC 25922 and *S. aureus* CECT 794 after a 4 h treatment with the AgNPs at 37 °C was evaluated by using the 2′-7′-dichlorodihydrofluorescein diacetate (Sigma-Aldrich, Co., St. Louis, MO, USA) (DCFH-DA) method following the protocols used by other authors [39] and us in previous studies [55,56]. The bacteria-killing activity was determined by colony counting of the remaining viable cells after the 4 h treatment, as in our previous work [55]. Briefly, 7.5 × 10^7^ CFUs/mL of the control bacteria in phosphate saline solution (PBS) were incubated in the presence of 5 µM DCFH-DA and increasing AgNP concentrations. Thereafter, the generated fluorescence was measured in a FLUOStar^®^ Omega plate reader using 485/520 nm for excitation/emission. After the fluorescence measurements, the AgNPs’ killing effect under these conditions was determined by counting the CFUs/mL obtained after plating 50 µL of 1/20,000 dilutions of the samples in nutritive agar.

### 2.7. Statistical Analysis

Statistical parameters—mean, median, and mode—of the AgNPs core diameters and the size distributions were calculated with Microsoft Excel for Microsoft 365 MSO version 2503.

The estimation of the IC_50_ and ICb_50_ values, the ROS accumulation analyses, and the evaluation of the statistical significance of the corresponding results were performed using GraphPad Prism 8 (GraphPad Software, San Diego, CA, USA).

A two-tailed unpaired *t*-test with a *p*-value of 0.05 allowed us to evaluate the statistical significance of the differences between the IC_50_ and ICb_50_ values of the AgNPs depending on the growth phases and the Cl^−^ content of the broths used in their synthesis.

For the statistical studies on ROS accumulation and bacterial viability in the presence of increasing concentrations of AgNPs, one-way ANOVA tests with a Dunnett’s multiple comparison and a *p*-value of 0.05 were used.

## 3. Results and Discussion

### 3.1. Phylogenetic Identification of the Bacterial Isolate

**Figure 1:**
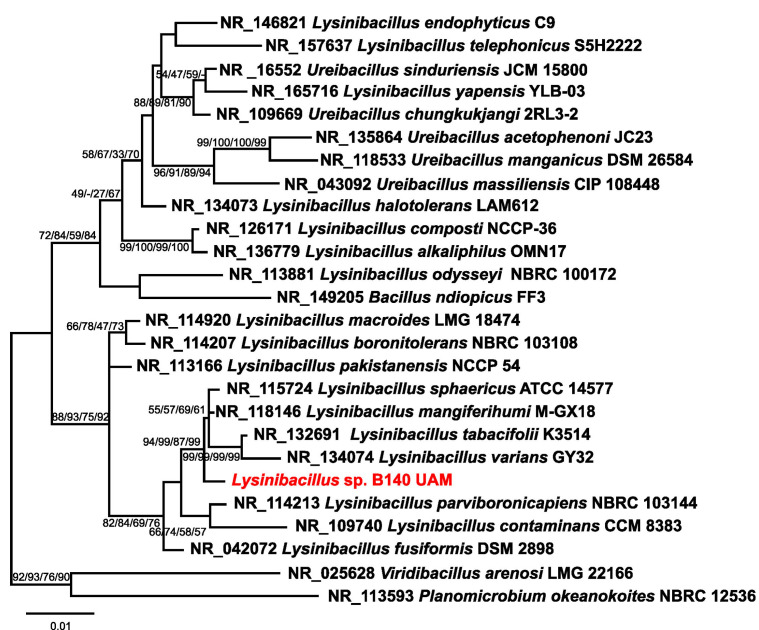
Phylogenetic tree for assignment of the isolate B140 UAM (in red) as a *Lysinibacillus* species. The tree structure shown is that of the neighbour-joining analysis, and the numbers in the branching points are the bootstrap values for, from left to right, the analyses using the maximum likelihood, minimum evolution, maximum parsimony, and neighbour-joining methods, respectively. The bar indicates the scale of the branches’ phylogenetic distances.

The microorganism used in this work for AgNPs production proceeded from our previous studies of the Tinto River microbial diversity [57,58]. After the corresponding phylogenetic analysis using the 16S rRNA sequence, compared to the closest species type strain sequences, it was ascribed to a species not previously described of the genus *Lysinibacillus*, based on the obtained phylogenetic tree in which the 16S rRNA sequence of the *Lysinibacillus* sp. BE140 UAM isolate appears in a clearly differentiated branch from other phylogenetically closed species of this genus (Figure 1).

Culture broths of this *Lysinibacillus* isolate were used for the extracellular synthesis of four types of AgNPs.

### 3.2. Growth of Lysinibacillus sp. in Two Culture Conditions

**Figure 2:**
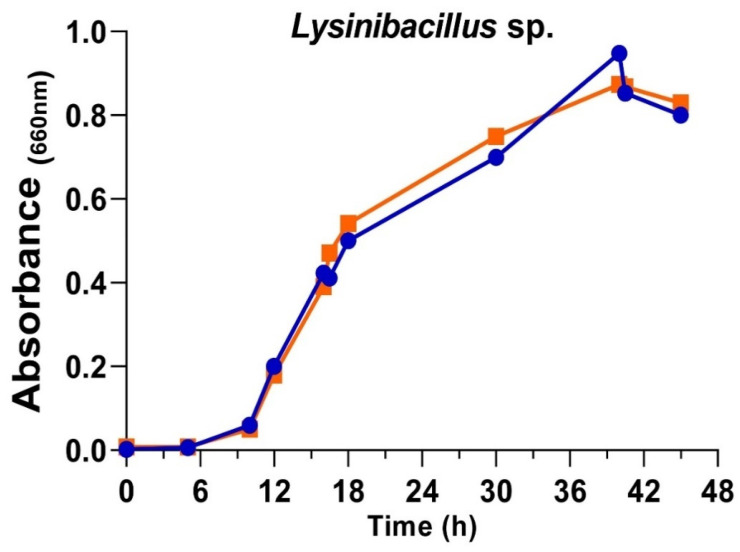
Growth curves of *Lysinibacillus* sp. in complete nutritive medium (blue) and medium without added NaCl (orange).

The presence of chloride in the culture medium of several organisms could affect their growth and the characteristics and properties of the AgNPs obtained from the corresponding culture broths [33,55,56,61]. For instance, the *Pseudomonas alloputida* used previously in preparing AgNPs [56] grew faster in media with chloride. However, the antibacterial activity of the AgNPs obtained with chloride-containing broths was consistently lower than that of those from broths without it. So, to cultivate the *Lysinibacillus* sp. isolate in the current work, media with or without NaCl were also used. No apparent differences in the growth rates and growth curve shapes were observed (Figure 2). These results agree with those described by Xu et al. [62], who observed that *Lysinibacillus* cultures reached their maximum growth rate at NaCl concentrations in the 0–2% range. In the current study, the cultures were set with NaCl at 0% and 0.5%, respectively, for the medium without or with chloride. The lag phase was around 6 h, with an exponential phase of 34 h. Broths used for preparing AgNPs came from the exponential and stationary phases of the cultures.

The growth phase of the cultures from which the broths are derived can affect both the composition and the synthesis kinetics of AgNPs and, consequently, their properties, as described in some previous studies on metal nanoparticles by other authors and us for a variety of microorganisms [55,56,63,64,65,66].

### 3.3. Synthesis of AgNPs Using Lysinibacillus sp. Culture Broths

**Figure 3:**
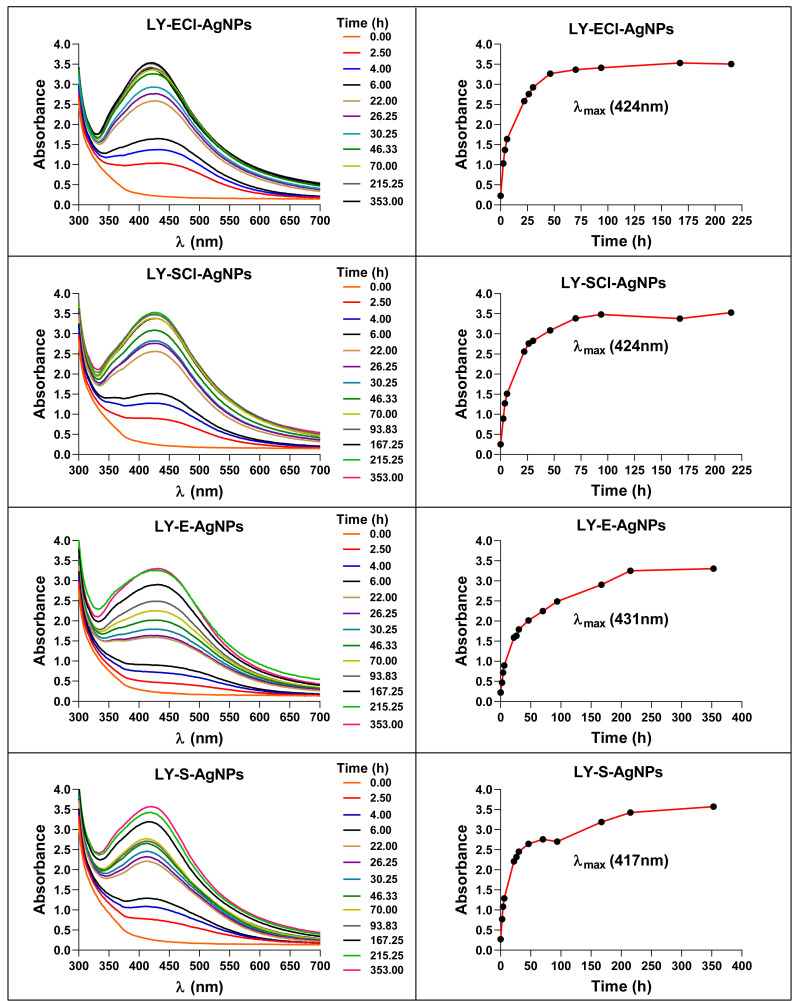
Changes in the UV-Vis spectra of the AgNPs during their synthesis. The right panels show the synthesis kinetics from the increase in absorbance at the corresponding λ_max_ (indicated in brackets).

From the bacterium cultures, cell-free broths were obtained and used for the AgNPs’ production from AgNO_3_. The synthesis kinetics followed by the UV-Vis spectra of the reaction mixtures (Figure 3) showed a time-dependent increase in absorbance, with a peak of maximum absorbance at wavelengths (λ_max_) of 424 nm for AgNPs from media with NaCl and 431 nm and 417 nm, respectively, from media without NaCl in exponential and stationary phases. Traditionally, these λ_max_ were related to the size and the size dispersion of the nanoparticles, associating the lowest value of λ_max_ with the smallest nanoparticles [67].

The maximum optical densities (OD_max_) reached by the AgNPs were very similar for the various AgNPs, but with slightly lower values for AgNPs from broths of exponential, with OD_max_ ≈ 3.25, and stationary phases, with OD_max_ ≈ 3.40.

Regarding the presence or absence of NaCl in the medium used for the cultures, there were no significant differences in the OD_max_ reached by the AgNPs. However, this variable affected the AgNPs synthesis rates, which were lower when using media with NaCl, maximum absorbance achieved between 48 and 72 h, than when media without NaCl were employed, reaching the highest absorbance at 215 h.

Other authors described that stationary phase broths were more efficient at synthesising AgNPs [63,68], while others observed a better rate with exponential phase broths [69]. Despite these discrepancies, previous results from our laboratory using different microorganisms showed similar results to those obtained here [55,56].

**Figure 4:**
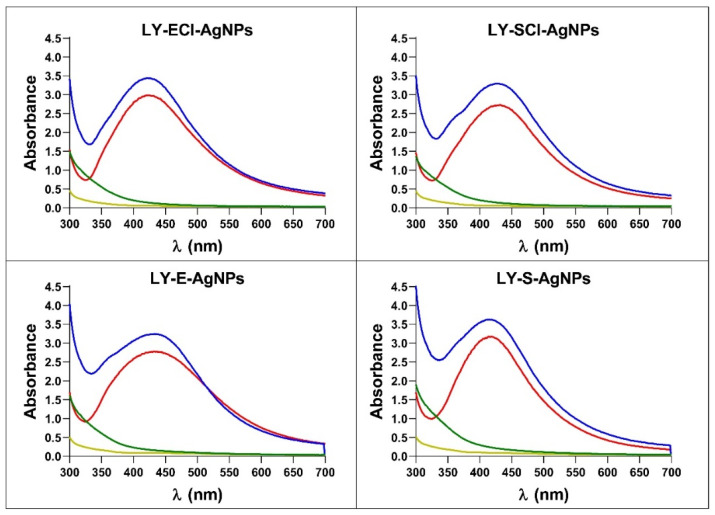
UV-Vis spectra of AgNPs, broths, and washing waters during the collection of AgNPs after their synthesis. In blue, AgNPs synthesis reactions; in red, AgNPs after washing; in green, supernatants after AgNPs collection; in yellow, Milli-Q water from the last wash.

When the synthesis reactions were left to proceed after the maximum production was reached, up to 175 h or 350 h, respectively, for AgNPs’ syntheses with broths with NaCl or without NaCl, no changes in the spectra and AgNPs production occurred. On the contrary, in our previous studies on the biogenic synthesis of AgNPs using other microorganisms, a decrease in absorbance at the λ_max_ and an increase at higher wavelengths (550 nm) indicated the appearance of aggregation processes [55]. Jalab et al. [70] also reported a similar aggregation when the synthesis reaction proceeded for a longer time. In addition, we showed previously, for biogenic AgNPs obtained with *Parachlorella* sp. cultures, that the longer the delay in collecting and washing the AgNPs after reaching the maximum production, the lower their antibacterial activity [55], so, in the current study, the collection of the AgNPs was carried out immediately after the AgNPs spectra reached the maximum absorbance. After the washing process, the AgNPs spectra showed no significant changes, indicating that the AgNPs retained their original structure during the collection and washing (Figure 4). The broths recovered during centrifugation after the synthesis and the washing waters showed minimal residual absorbance, indicating that the procedure recovered most nanoparticles from the reaction mix.

The favoured synthesis of AgNPs in media containing NaCl could be due to a different organic composition of the broths, resulting from better bacterial growth in this medium. Such differential growth in cultures with or without NaCl was observed in previous studies [55,56] that seemed to be related to the differential rate of AgNPs’ synthesis also observed. However, no such bacterial growth difference appeared in the current study, but a difference in AgNPs synthesis rate remained. Moreover, Gurunathan et al. described the absence of *Escherichia coli* differential growth in media with or without NaCl, with a difference in the rate of AgNPs synthesis, using the corresponding broths, higher for those with NaCl [68], as happened in our current study. Thus, the absence of changes in the growth curves in the two conditions does not indicate that the media have the same composition and capabilities for AgNP production. Another factor that could be involved in the synthesis rate differences observed could be the very presence of Cl^−^ in the medium, as various authors have suggested [33,55,56,61,71]. The presence of this anion would produce the precipitation of some of the silver as AgCl that could serve as nucleation points for AgNP formation, generating higher synthesis rates.

### 3.4. Stability of the AgNPs in the Dark at 4 °C

**Figure 5:**
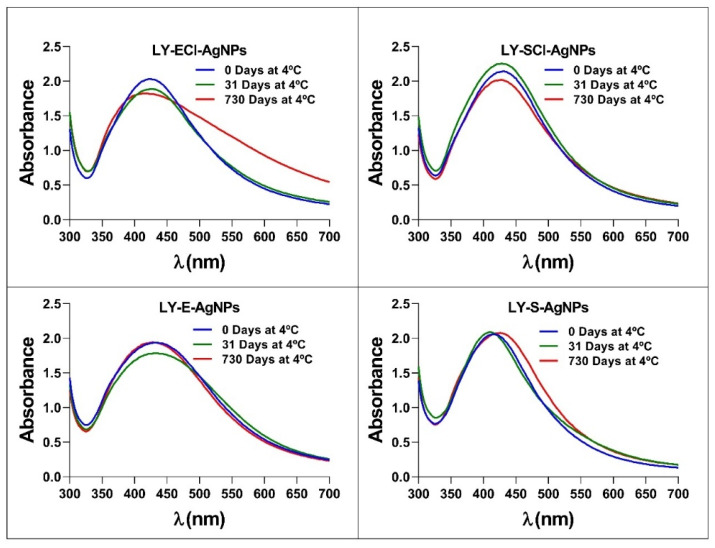
UV-Vis spectra of AgNPs after storage in darkness at 4 °C for different periods.

Analyses determined the possibility of preserving the AgNPs at 4 °C and in the dark concerning their structural stability and antibacterial activity. The UV-Vis spectra registered after different periods showed no changes initially up to one month after the AgNPs’ collection. The experiment followed up to 2 years, and only the LY-ECl-AgNPs showed an increase in absorbance at λ_550_, indicating some aggregation of the AgNPs after this very long time (Figure 5). Additionally, the MIC values against *E. coli* for each AgNP kept under these conditions during the different periods remained unchanged, as shown in Table S1. In a previous study, we showed other AgNPs to be stable for up to at least two weeks of conservation in the same conditions [56]. Other authors also observed this behaviour for chemical AgNPs kept at 4 °C for 78 days without affecting their spectra or even for a year with minimal variation compared to the same AgNPs kept at room temperature [72]. Moreover, Pham [73] also used the conservation of AgNPs at 4 °C, and Dede et al. [74] mentioned that the AgNPs produced in their work retained their properties for one year, although they did not mention the preservation method. Finally, previous work from our laboratory showed that biogenic AgNPs kept at 4 °C for ten months in the light retained their spectra and most of their antibacterial activity. In contrast, those kept at 30 °C ceased to be active when analysed after the same time [33]. All these data indicate that biogenic AgNPs can retain their properties, like chemical AgNPs, for a certain period when refrigerated at 4 °C, which facilitates their use. The current study is the first of this kind to test the stability of biogenic AgNPs for very long periods, showing that most of the tested biogenic AgNPs can be conserved in the dark at 4 °C, with no or only a slight effect on their aggregation.

### 3.5. Physicochemical Characteristics of the AgNPs

#### 3.5.1. AgNPs’ Elemental Composition Determined by Total Reflection X-Ray Fluorescence (TXRF)

**Figure 6:**
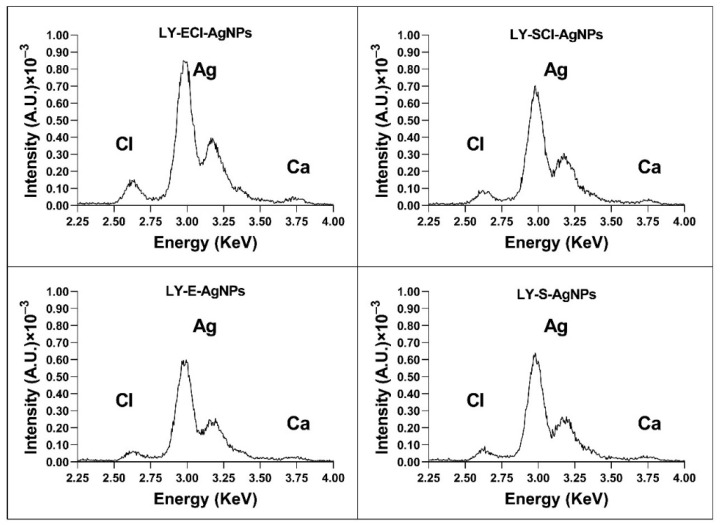
TXRF analysis of the elemental composition of AgNPs around the silver peaks.

The TXRF analysis showed that silver was the main component of all the nanoparticles, appearing as two characteristic peaks in the 3–3.25 KeV region (Figure 6). Signals corresponding to other elements also appeared in very low concentrations. Of particular interest in this study is the peak located in the 2.6 KeV region, which corresponds to chlorine (Figure 6). This element appeared in all the nanoparticles, but in higher concentrations in the AgNPs prepared with broths from cultures in the regular nutritive medium (containing NaCl). The appearance of low amounts of chlorine in the AgNPs prepared with a nutritive medium with no added NaCl could be due to the presence of this element in trace amounts in the components used to make up the medium. However, in our previous studies [55,56], we observed slightly higher differences in chlorine concentration between those prepared with broths with or without added NaCl than in the current study. Moreover, other authors used media with [75] or without chlorine [50] to synthesise AgNPs, and this element appeared in the AgNPs from the medium with it. However, in other studies, the chlorine concentration of the culture media and the chlorine presence in the corresponding AgNPs did not correlate. For example, Abd Alamer et al. obtained silver nanoparticles containing a high concentration of chloride (they called them AgCl-NPs, indicating this fact) by using a broth in which chlorine concentration was 1.75 g/L [76], lower than in the regular nutritive medium (5 g/L). Moreover, Bhatia et al. synthesised AgNPs using the broth diluted at 50% from a bacterial culture in basal salt medium, with a 0.5 g/L NaCl concentration, and obtained AgNPs showing a high Cl^−^ content via dispersive X-ray spectroscopy (EDX) analysis [51]. On the other hand, in our current work, with only a 5% dilution of the chlorine content of the broths (a 4.75 g/L concentration of NaCl in the chloride-added broths), the concentration of chlorine in the AgNPs measured by TXRF is considerably low (Figure 6). These observations reinforce our previously published hypothesis [55] that the presence of chlorine in the AgNPs does not depend only on the chloride concentration of the broths used for their synthesis but also on the organic composition of the broths that may affect the deposition of chloride during the AgNPs formation, also in agreement with our current results.

We consider that the presence of this element in the nanoparticles’ composition plays a relevant role in the antimicrobial properties of these materials, as our previous studies have suggested [33,55,56]. The knowledge of the proportion of chloride in the AgNPs and their silver concentration is quite relevant for determining the activity of the AgNPs in a comparative way with other studies. However, some studies do not control this characteristic of the AgNPs [52,77,78], making activity comparisons difficult or impossible.

#### 3.5.2. AgNP Crystallinity Determined by X-Ray Diffraction (XRD)

**Figure 7:**
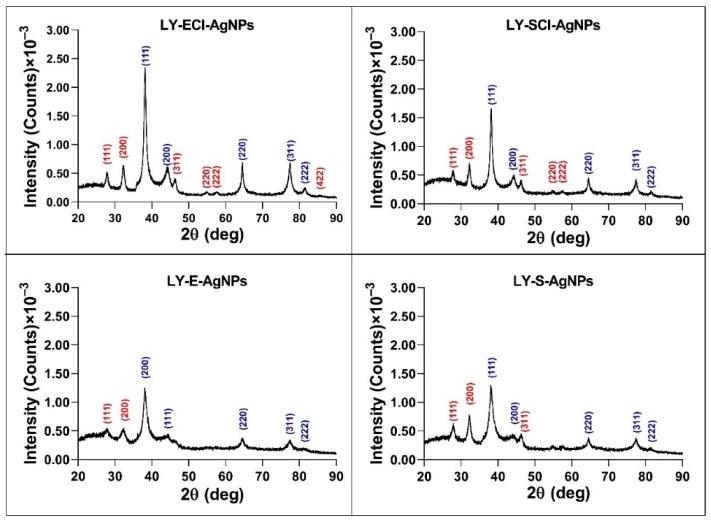
XRD patterns of the AgNPs. Planes of Ag^0^ (blue) and AgCl (red) crystals are indicated.

The XRD analysis showed peaks with 2θ values (in degrees) around 38.18, 44.34, 64.56, 77.50, and 81.50 (Figure 7), corresponding to metallic silver crystals (planes (111), (200), (220), (311), and (222), respectively, of their face-centred cubic structure (Joint Committee on Powder Diffraction Standards (JCPDS) file 04–0783)). Additional peaks with 2θ values (in degrees) of 46.26, 54.88, 57.48, 67.48, 74.50, 76.80, and 85.80 appeared in AgNPs from media with NaCl, corresponding to the (220), (311), (222), (400), (331), (420), and (422) planes, respectively, of the cubic crystalline structure of AgCl (JCPDS file 31–1238). The peaks corresponding to AgCl comprised a smaller relative area than those of the Ag^0^, indicating a higher proportion of metallic silver crystals in these nanoparticles and a lower proportion of AgCl crystals.

When comparing our results with those obtained for AgNPs in previous studies, the patterns were very similar to those obtained for AgNPs from *P. alloputida* cultures, although showing more prominent AgCl peaks in media without added NaCl than in the AgNPs from *P. alloputida* cultured in the same medium [56]. In the case of the AgNPs prepared with culture media from *Parachlorella* sp., in a completely different medium containing a lower chloride concentration than the nutritive medium, the peaks corresponding to AgCl crystals were more prominent [55]. So, the results obtained in the current study also reinforce our previous hypothesis that the chloride concentration in the broths used for silver nanoparticle synthesis does not determine the presence of more or fewer AgCl crystals in the AgNPs.

The XRD analyses of the crystallinity of AgNPs obtained by other authors using *Lysinibacillus* cultures have shown the presence of metallic silver crystals alone [50], but in other cases [49,51,52], peaks associated with AgCl crystals have also appeared, even more prominent than in our AgNPs prepared with broths containing NaCl. Omole et al. explained that these peaks might be due to the presence of organic compounds in the AgNPs, even though their EDX results have shown that chlorine was one of the most dominant elements, together with silver, in the composition of their AgNPs [49]. Moreover, some other reports have described the presence of these crystals in AgNPs of biogenic origin [75,76,79], omitting their assignment to AgCl crystals, in some cases as the major component of the nanoparticles versus those of Ag^0^ [76,80].

#### 3.5.3. The Shape and Size of the AgNPs Core Determined by Transmission Electron Microscopy (TEM)

**Figure 8:**
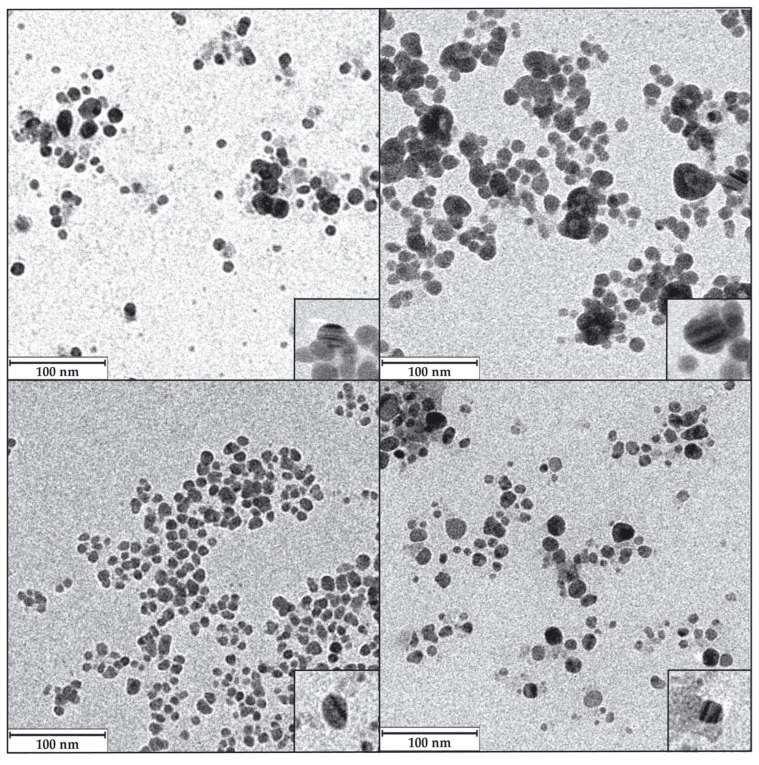
Representative TEM images of the AgNPs. LY-ECl-AgNPs (**upper left**); LY-SCl-AgNPs (**upper right**); LY-E-AgNPs (**lower left**); LY-S-AgNPs (**lower right**). Down-right corner insets in each panel show an amplified image of a nanoparticle with internal parallel patterns. Magnification for the images was 1.5 × 10^5^, and for the insets, it was 1.2 × 10^6^.

An analysis of the AgNPs TEM images allowed us to determine the shape and average size of the AgNPs’ metallic cores. The nanoparticles obtained in this study were, generally, quasi-spherical, with a minority of other morphologies (Figure 8). AgNPs produced by different biological methods have shown, as in the current case, the predominance of quasi-spherical forms, whether synthesised by *Lysinibacillus* cultures [49,51,54], other bacteria, or different microorganisms [3,10,11]. Despite this, several articles and reviews have established a variety of AgNP shapes, which may be related to the methodologies and materials used in their synthesis, so that by varying them in a controlled manner, nanomaterials of one shape or another could be obtained [81,82,83,84].

Some authors have suggested that the AgNPs’ shape can influence their antibacterial activity. Thus, triangular shapes are claimed to be one of the most active ones, assuming that their high number of faces favours the release of Ag^+^ ions and their interaction with the bacteria, releasing more ionic silver and also producing perforations of the membrane by their sharp vertices [15,85]. Despite all this, other authors have presented data in which spherical AgNPs presented better efficacy than triangular ones and considered that this was due to a better release of Ag^+^ ions because of their higher surface-to-volume ratio [82,86]. Finally, a recent review stated that the most relevant issue in relating the antimicrobial activity of AgNPs and their shape is their surface area in contact with the microorganism [15], giving preference to spherical, triangular, and octahedral objects over more elongated ones, such as bacillary shapes or nanotubes. The quasi-spherical AgNPs presented in this paper showed excellent antibacterial activity, as will be described later in this report.

The TEM images also recorded different levels of aggregation of the AgNPs, with the most aggregated ones coming from media with Cl^−^, as also happened in previous works [49,55,56]. The images also showed internal structures with differentiated density zones within some AgNPs, appearing as alternating darker and lighter lines (insets in Figure 8). Some authors have seen these same structures, which were claimed to be typical of AgClNPs [76,87]. However, the presence of these structures in AgNPs whose XRD does not show prominent AgCl peaks, as in some studies [33,55,56,88,89] and in the one presented here, contradicts the possibility of only associating them with AgClNPs. In our opinion, the bands of different intensities could reflect layers of crystals with different orientations, which could be presented to the electron beam of the microscope with a higher or lower density of atoms depending on their orientation on the TEM mesh grids; however, the reason for the formation of such crystals layers with different orientation is unknown.

**Figure 9:**
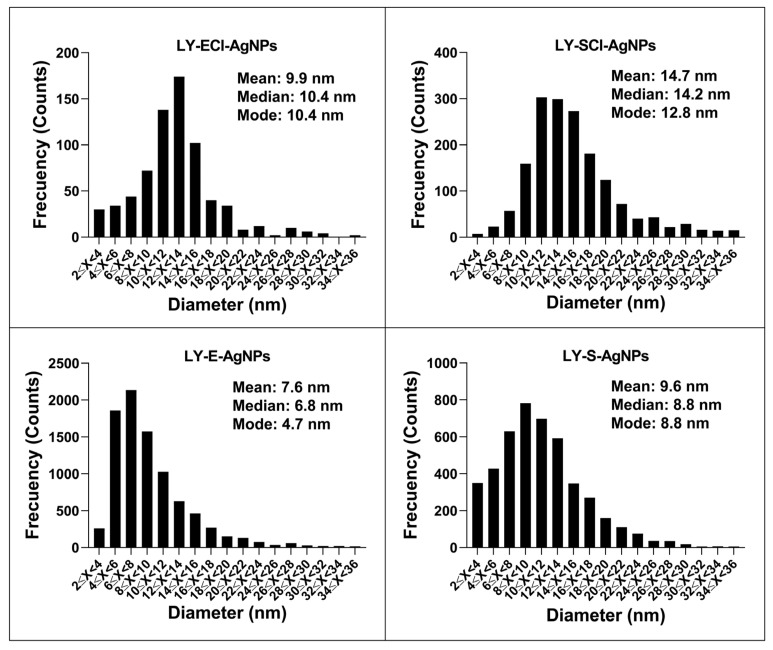
Distribution of AgNPs’ core diameters grouped in two nm ranges.

The distribution of the AgNPs’ core sizes was different depending on the type of AgNPs (Figure 9, Table 2), and their average diameter ranged from 7.5 to 9.9 nm for all AgNPs except for LY-SCl-AgNPs, which had a larger average diameter of 14.7 nm. The AgNPs from media with NaCl showed a larger average diameter than those without this salt. Additionally, the AgNPs from stationary phase cultures were bigger than their counterparts from exponential phase cultures, especially those produced from chlorine-containing media. Similarly, the polydispersity indices were below 0.40 for most AgNPs, indicating a moderate size dispersity [90,91], although the LY-E-AgNPs presented a higher dispersity (PDI = 0.50). Similar correlations were observed for the AgNPs from *P. alloputida* [56] and *Parachlorella* [55] obtained in previous studies.

In general, the average sizes of the AgNPs synthesised in this work were considerably smaller compared to those of other AgNPs that were synthesised from different microorganisms [10,11] and specifically from cultures of other *Lysinibacillus* species, such as those obtained by Liu et al. with *L. fusiformis* [19] and *L. sphaericus* [54], which have shown sizes of 50 and 40 nm, respectively. However, AgNPs prepared by other authors with cultures of different strains of the same species were smaller, 18.42 nm for those from *L. fusiformis* [49] and 14–21 nm for other AgNPs obtained with *L. sphaericus* cultures [53]. Other authors have also succeeded in producing AgNPs from other *Lysinibacillus* species, with sizes ranging from 8 to 30 nm [50,51,52]. This variety of sizes could be due to the different bacterial species and strains, as well as the various culture media and synthetic methodologies used.

#### 3.5.4. Hydrodynamic Size and Z-Potential of the AgNPs Determined by Dynamic Light Scattering (DLS) and Electrophoretic Light Scattering (ELS)

The hydrodynamic sizes of the AgNPs (Table 2, Figure S2) were very close for AgNPs from media with NaCl (LY-ECl-AgNPs and LY-SCl-AgNPs), with diameters of 48.5 nm and 52.7 nm, respectively, but more different for the AgNPs from media without NaCl (LY-E-AgNPs and LY-S-AgNPs), with diameters of 80.2 nm and 63.3 nm, respectively. Moreover, the AgNPs from media without NaCl had the highest hydrodynamic diameters of all the AgNPs prepared in this work, presenting a higher difference with the corresponding metallic core diameters, suggesting thick coronas. Other authors also observed a considerable increase in the size of AgNPs when comparing the core and hydrodynamic sizes, obtaining similar conclusions to those obtained in this work [19,49,51,52,54].

The Z-potentials of the AgNPs were all negative and ranged from −20.6 to −25.1 mV, with only LY-S-AgNPs showing a notably different value of −8.8 mV. No clear relationship appeared between the Z-potential values due to the culture phase or the presence or absence of NaCl in the media used for the AgNPs’ synthesis. Some authors have claimed that this parameter is related to the stability of the AgNPs, considering them more stable when this is high due to the higher repulsion between nanoparticles with the same charge sign [92], but others have disagreed [93]. Previously described guidelines [93,94] classify nanoparticle dispersions with Z-potential values of ±0–10 mV as unstable and ±20–30 mV as stable. In this sense, most AgNPs obtained in this work should be considered stable, except LY-S-AgNPs. However, no other data obtained in this study would justify classifying these last AgNPs as unstable. AgNPs obtained by others from *Lysinibacillus* also showed some variations in the Z-potential values [19,51,52,53].

#### 3.5.5. Insights into the AgNPs’ Corona Composition Determined by Fourier Transform Infrared Spectroscopy (FTIR)

**Figure 10:**
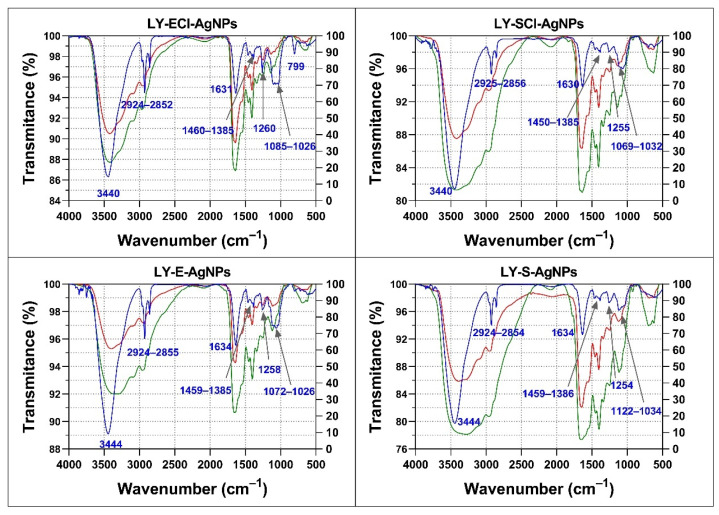
FTIR analysis of the AgNPs’ organic composition and broths. In blue, AgNPs’ spectra; in green, broths before synthesis; in red, broths after synthesis. The wavenumbers of the principal AgNPs’ bands are indicated.

An FTIR analysis of the organic components of the AgNPs, the broths used in their synthesis, and the broths after their use was carried out (Figure 10). The FTIR spectra of the broths before and after synthesis showed insignificant differences, which indicated that few of their components got attached to the AgNPs’ cores.

The spectra of the broths and the AgNPs obtained with them showed some differences. For instance, the broths showed many bands in the 1630–1030 cm^−1^ region and low transmittance values for the broad band ranging from approximately 3500 cm^−1^ to 2800 cm^−1^. On the contrary, the spectra of the AgNPs were simpler, showing only a small group of bands, which indicated that not all organic components present in the broths ended up forming the corona of the AgNPs.

All the AgNPs showed spectra with a broad band in the 3440–3444 cm^−1^ region assigned to -OH and -NH of amide groups, a triplet signal around 2852–2956 cm^−1^ associated with aliphatic groups, a band around 1630–1634 cm^−1^ associated with -C=O of amide I and II, three subregions in the fingerprint region corresponding to 1385–1460 cm^−1^ also associated with -C=O of amide I, a single band at 1254–1260 cm^−1^, and a triplet around 1026–1122 cm^−1^ associated with -C-O-C of carbohydrates (Figure 10). The assignment of infrared bands to specific molecules is speculative in the case of complex mixtures, such as those present in the broths used for the obtention of AgNPs and in the obtained nanomaterials. In these cases, the analysis is limited to the types of biomolecules or their relative proportions [95,96].

Comparing the spectra of the various AgNPs, all showed bands at approximately the same wavenumbers, but their relative transmittances were different in some cases, mainly in the fingerprint region (1300–400 cm^−1^). In this regard, AgNPs from stationary phase cultures showed a lower transmittance of the band around 1640 cm^−1^ compared to the triplet around 1060 cm^−1^, relative to those of their exponential phase AgNPs counterparts, which could indicate a composition with a relative higher presence of carbohydrates in LY-ECl-AgNPs and LY-E-AgNPs coronas in comparison to their counterparts obtained from the stationary cultures. Moreover, the bands at 2852–2956 cm^−1^ showed higher transmittance compared to the 3400–3444 cm^−1^ band in AgNPs from stationary phase cultures compared to those from exponential phases, which could indicate the higher proportion of biomolecules with aliphatic groups in the coronas of the former.

Other authors have also detected the presence of several different components in the corona of biogenic AgNPs with differential bands for different AgNPs using FTIR, suggesting differences in the coronas’ composition. For instance, Huq et al. observed very pronounced bands in the 2359 and 2342 cm^−1^ regions, which they associated with the presence of alkyne groups [50], and Bhatia et al. also observed peaks at 1288 cm^−1^ associated with -CN amide groups and -NH from peptide bonds [51,52]. The study of the biogenic AgNPs’ coronas composition by FTIR does not unequivocally determine the specific compounds present in them. The FTIR assays can determine or confirm the presence or absence of such compounds only for AgNPs obtained using unique compounds or low-complexity mixes.

### 3.6. AgNPs’ Antibacterial Activity

The use of the microdilution method allowed us to perform a quantitative assessment of the growth of the test bacteria in the presence of several AgNPs concentrations to calculate the antibacterial parameters: minimum inhibitory concentration (MIC), minimum bactericidal concentration (MBC), 50% growth inhibitory concentration (IC_50_), and 50% biofilm formation inhibitory concentration (ICb_50_) (Table 3, Tables S1 and S2). These studies included the antibiotic streptomycin (Sm) and silver nitrate (AgNO_3_) as references to compare with the AgNPs’ antibacterial efficacies.

The antibacterial activities of the AgNPs against the different test bacteria (Table 3) were, in all cases, higher than that of streptomycin. Furthermore, the strain of *S. epidermidis* used as one of the test bacteria in this study was resistant to this antibiotic, and growth inhibition with streptomycin was not achieved even at concentrations up to 256 µg/mL. However, inhibition was possible using ionic (AgNO_3_) or elemental (AgNPs) silver. These results showed that the AgNPs produced in this work were effective inhibitors of bacterial growth in all the tested bacteria, including the streptomycin-resistant strain of *S. epidermidis*, at concentrations lower than those required for streptomycin. However, in all cases, the antibacterial activity of AgNPs did not reach the levels achieved by the ionic silver (AgNO_3_). The AgNPs obtained by Pietsch et al. also showed less activity against *P. aeruginosa* [97] than AgNO_3_, but more than the antibiotic. Some other studies have also supported the higher activity of silver ions over AgNPs [98,99]. An explanation for this behaviour could be that both AgNPs and Ag^+^ are supposed to be able to interact with many bacterial components and also with biofilms [100], although other studies have shown that the obtained AgNPs were more effective than Ag^+^ [101,102]. The results of our previous studies have shown that most of the AgNPs obtained from *P. alloputida* [56] and *Parachorella* sp. [55] cultures were less effective than AgNO_3_, but still with some exceptions of AgNPs more active than the ionic silver, depending on the control bacteria tested. These differences indicate that either situation is possible depending on the AgNPs and, in some cases, the species or strain used for antibacterial activity testing.

The AgNPs’ MIC values obtained in this work were between 0.32 and 9.05 µg/mL, with some differences depending on the AgNPs’ characteristics and the bacterial species tested. The AgNPs were more effective against the Gram-negative bacteria, with MICs in the range of 0.32–2.26 µg/mL, *P. aeruginosa* being particularly susceptible. Another strain of this species has also been very susceptible, as described by Dove et al. [103]. This fact is relevant, as the scarcity of antibiotics effective against this type of bacterium due to their antibiotic resistance led the World Health Organisation (WHO) to assign the highest priority to the search for antibacterial agents against Gram-negative bacteria [104,105]. The Gram-positive bacteria tested were also susceptible to AgNPs but required, in some cases, using higher concentrations (MICs 1.26–9.05 µg/mL). Among these, *S. aureus* was the least susceptible to the antimicrobial action of silver, both in ionic form and as AgNPs. Other authors have found AgNP resistance in Gram-positive bacteria of the *Staphylococcus* genus [106]. In some other studies, the same general pattern of AgNPs’ activity appeared, in which two AgNPs with different morphology showed better activity against Gram-negative bacteria than against Gram-positive ones [107], including a higher susceptibility of *P. aeruginosa* and a higher resistance of *S. aureus*. In addition, a recent article concluded, after analysing a literature review, that the antibacterial activity of AgNPs was generally better against Gram-negative bacteria [108], attributing this to structural differences in the bacterial wall. However, this would depend on the AgNP type and the bacterial species or strains tested, as some studies have also reported the opposite [109,110,111].

Besides their good antibacterial activity, the MBC/MIC ratios of AgNPs were, in most cases, <4, which indicates bactericidal activity, according to previous studies [112]. This behaviour is not uncommon, as other recent studies have also claimed a bactericidal capacity of their AgNPs [108,113]. Nevertheless, some AgNPs studied by others presented a bacteriostatic effect [108,114]. In our study, AgNPs and AgNO_3_ presented a bacteriostatic mechanism against *S. epidermidis* and for specific AgNPs, such as LY-ECl-AgNPs against *P. aeruginosa* or *K. pneumoniae* or LY-E-AgNPs against *B. subtilis*. The level of antibacterial activity of the AgNPs, their mechanism of action, and the characteristics of the bacteria themselves probably play a role in whether the mechanism produced is bactericidal or bacteriostatic.

Another parameter used to measure the inhibition of bacterial planktonic growth, besides the MIC, is the IC_50_ (Table 3). Values of IC_50_ ranged from 0.08 to 0.61 µg/mL against Gram-negative bacteria and 0.42 to 3.24 µg/mL against Gram-positive ones. Moreover, the measurement of the ICb_50_ allowed us to determine the capability of AgNP and AgNO_3_ to inhibit biofilm formation (Table 3). The ICb_50_ values against the tested Gram-negative bacteria ranged from 0.18 to 1.11 µg/mL and were 0.92–5.20 µg/mL against the Gram-positive ones. Even though both activities were better against Gram-negative bacteria, the IC_50_ and ICb_50_ values were also low against the Gram-positive ones, indicating the excellent capability of these nanomaterials to prevent biofilm formation. Biofilms are structures considered resistant to the action of different substances, including antibiotics, and, in some cases, have a role in the antibiotic resistance of pathogenic bacteria [10,115]. Moreover, the ICb_50_ values were, in most cases, higher than the corresponding IC_50_, indicating that most of the synthesised AgNPs were more effective in inhibiting planktonic bacterial growth than biofilms. In some specific cases, the IC_50_ and ICb_50_ values were very similar, as for LY-SCl-AgNPs and LY-S-AgNPs against *P. aeruginosa* and *B. subtilis*, LY-S-AgNPs against *E. coli*, LY-ECl-AgNPs against *K. pneumoniae,* and LY-E-AgNPs against *S. epidermidis*. The LY-E-AgNPs against *E. coli* and *B. subtilis* showed lower ICb_50_ values than the corresponding IC_50_ ones, indicating a better activity of these AgNPs against biofilm formation than against planktonic cell growth. Some authors showed that some biogenic nanoparticles can be good anti-biofilm agents, as reviewed in [100], either alone or in combination with other compounds, as reviewed in [116]. This activity could be due to a multitude of factors, either because the characteristics of AgNPs allow them to attack already-formed biofilms or from the beginning of their formation in a preventive manner [116], sometimes inhibiting the formation of these structures by acting at the level of bacterial quorum sensing [100,117].

We used the IC_50_ and ICb_50_ parameters to compare the antibacterial activities of the different AgNPs in terms of their characteristics and origin. The culture’s growth phase used to produce the AgNPs significantly affected the antibacterial activity of the AgNPs.

Among the AgNPs prepared using NaCl-containing broths, those from the stationary phase were more active than those from the exponential phase against planktonic cells and biofilms of *E. coli* and *P. aeruginosa* and against planktonic cells of *S. aureus* and biofilms of *B. subtilis*. This higher efficacy of AgNPs from stationary cultures is curious since it contradicts the widespread idea that smaller AgNPs are more effective in producing an antibacterial effect [116,118], given that the LY-SCl-AgNPs have both larger core sizes, measured using TEM, and larger hydrodynamic diameters, measured using DLS, than the LY-ECl-AgNPs. The Z-potential would also not explain this difference in activity, as other authors have claimed [3,119,120], since both AgNPs presented very similar values (−20.63 and −21.58 mV). The only AgNPs feature that could better differentiate them is the organic composition of the corona, as the FTIR spectra of LY-SCl-AgNPs contain a lower proportion of carbohydrates and a higher proportion of aliphatic chains and proteins than those from the exponential phase. Some researchers observed significant antimicrobial efficacies of biogenic AgNPs, whose FTIR spectra showed high proportions of proteins in the coronas [49,51,53], but there is still no clear evidence of a high proportion of specific organic components in the corona to induce higher efficacy in the AgNPs.

On the other hand, among the AgNPs from broths without added NaCl, those from the exponential phase were more active against planktonic cells of *S. aureus* or *B. subtilis* and *S. aureus* biofilms than the ones from the stationary phase. LY-S-AgNPs were more active than the LY-E-AgNPs only against the *S. epidermidis* planktonic cells. Previous results of our research group with AgNPs produced with *P. alloputida* cultures showed similar conclusions [56]. The particular behaviour concerning the antibacterial activity of the LY-S-AgNPs could be related to its low Z-potential (−8.85 mV), since some authors have considered that this activity is much related to this parameter [121], but the influence of this parameter on the activity is not always in agreement with the experimental results.

Regarding the presence of NaCl in the broth used for the AgNPs synthesis, generally, the AgNPs from NaCl-containing media presented higher activity than those from media without added salt against all control bacteria except *E. coli*. The same results were obtained using AgNPs prepared with media from microalgae cultures [55], but contrary to those from *P. alloputida* [56]. Few articles have addressed the study of the relevance of this factor on the silver nanoparticle activity. However, Suchomel et al. compared the activity of AgNPs and AgBrNPs, finding better activity of the latter containing silver halide [122], and a recent review discussed the effect of the NaCl concentration in the synthesis and activity of silver nanoparticles, indicating that the presence of this salt induces the formation of AgCl crystals that favour its activity [38].

Since the *P. aeruginosa* strain used for antibacterial activity determination showed high susceptibility to the AgNPs, we wanted to check whether this characteristic was due to the strain used (CECT 108) or was a characteristic of the species, so two more strains (PA01 and PA14) of the same species were evaluated. The results indicated that *P. aeruginosa* was especially susceptible to silver in the two forms tested here, as the MICs and the rest of the antibacterial parameters analysed of the tested AgNPs (LY-S-AgNPs, with intermediate efficacy) against all three strains were very low and alike (Table S2). Similar results have been previously reported for other AgNPs [55,56,97,123], so this seems to be a characteristic behaviour of AgNPs against the *P. aeruginosa* species.

### 3.7. Synergistic Antibacterial Activity of the AgNPs with Classical Antibiotics

Another method of using AgNPs to fight bacterial infections is in combination with classical antibiotics [103]. This system not only allows lower concentrations of both antibiotics and AgNPs to be used effectively, reducing the possibility of reaching concentrations that produce cytotoxic or harmful effects in human cells [124], but could also allow for the recovery of antibiotics that are no longer in use due to their lack of efficacy on their own at safe concentrations or due to the generation of antibiotic resistance by bacteria [60,106,124,125,126,127,128].

In the studies that have been reported in the literature on the putative synergy between AgNPs and classical antibiotics, several methods, including the chequerboard, the zone of inhibition (ZOI), and the MIC fold, among others, have been used [10,20,42,129]. In our opinion, the most advisable method is the chequerboard method, based on the fractional inhibitory concentration index (FICI). This parameter allows for the quantification and the use of a scale to determine whether synergy exists [44,45]. However, several authors or institutions have used different FICI scales to interpret its value. The main differences concern the use of the term “partial synergy” that some authors have considered when the FICI is 0.5 < FICI ≤ 1, which others have considered inconsistent with the synergy concept [44] and thus should be avoided. Another discrepancy in the scales is that some authors, following the EUCAST recommendations [130], have considered an antagonist behaviour for FICI > 2, while other authors have required a FICI > 4 to apply this term [45]. To decode the results of our synergy studies, we will consider the scale stated in Section 2.5. The most commonly used method for studying AgNPs-antibiotic synergy measures the size of the inhibition zone (ZOI) produced on semisolid nutrient media when antibiotic and AgNPs, incorporated together into a paper disk, are placed onto the plate surface previously inoculated with a test bacterium or in a hole, and after some incubation time. In this method, the synergy assessment is performed based on the ZOI increase produced by the AgNPs-antibiotic combination over the ZOI generated by the antibiotic alone. However, this method has many drawbacks that do not lead to the correct interpretation of the data obtained, with no established scale for the ZOI increase that would allow us to discriminate whether there is synergy or not. Moreover, different calculations from the measured inhibition halo sizes have been performed depending on the authors. The interpretation of the size changes of the inhibition zones in many studies using this method is that any increase in the halo would indicate a synergy, without considering the inhibition zone produced by the AgNPs alone. Some authors have enumerated various problems this method presents [42,43]. Arsene, in a clarifying paper, proposed modifying the calculations that most authors perform to ameliorate the interpretation of the results [43]; however even with this, the main problems of the method, which are intrinsic to it, such as the differences in the diffusion rate of the AgNPs into the agar or the lack of knowledge of the working concentrations, cannot be fixed. We will discuss our results in relation to studies that have used the FICI since this will allow us to make significant comparisons with our data. We will comment on the published results using other methods when no report using the FICI exists.

The synergistic behaviour of our AgNP (or AgNO_3_)-antibiotic combinations was evaluated by determining the FICI and the modulatory factor (MF) [44,60] (Table 4), finding different levels of effect of the AgNPs on the antibiotic MICs depending on the AgNPs (Table S3), the antibiotic, and the bacteria tested, as other authors have previously observed using various bacterial species [106,131,132] and diverse strains [106,133,134,135].

When we tested the various AgNP types synthesised in this work and silver ions (AgNO_3_) against *E. coli*, synergy appeared for any of them with chloramphenicol (FICI = 0.188–0.375 and MF = 8–4) or rifampicin (FICI = 0.094–0.188 and MF = 16–8). All the AgNPs also synergised with tetracycline (FICI = 0.310–0.500 and MF = 16–4), but there was no synergy for AgNO_3_-antibiotic combinations. Most of the AgNPs and the AgNO_3_ worked synergistically with vancomycin (FICI = 0.375 and MF = 8–4), but not the LY-ECl-AgNPs (FICI = 0.625 and MF = 2), and only the LY-SCl-AgNPs and LY-S-AgNPs acted synergistically with erythromycin (FICI = 0.500 and MF = 4), while synergy with ertapenem (FICI = 0.500 and MF = 4) was limited to the LY-S-AgNPs.

The FICI values were higher for the combinations with the AgNO_3_ than the AgNPs for 42% of the combinations and the same for 35%. The synergy was stronger with the AgNO_3_ than with the AgNPs for only 22.8% of the combinations. The synergy with colistin or kanamycin was higher for all the AgNPs than for AgNO_3_. The same happened for tetracycline, but synergy of AgNO_3_ with this antibiotic was not detected. With chloramphenicol or rifampicin, the AgNO_3_ and the LY-s-AgNPs synergised at the same level, and synergy was stronger with the other AgNPs. As far as we know, very few papers have described comparisons of the antibacterial activity of AgNPs and AgNO_3_, and even fewer in combinations with antibiotics, as in [136,137], who have used different test bacteria than in our study. In these cases, both AgNO_3_ and AgNPs produced synergic effects, with similar behaviour for both forms of silver. The more dissimilar behaviour we observed in our study for synergy with both silver types was that of tetracycline, for which the AgNPs but not the AgNO_3_ showed synergy. Thus, this may open the possibility of new comparative studies on the differential behaviour of the ionic silver and the AgNPs as antibacterial agents and in combination with antibiotics, by using our AgNPs. Comparing the level of synergy observed for AgNO_3_ and the AgNPs, we saw three situations, as stated above. We can hypothesise that when the AgNPs produce the same level of synergy as the ionic silver, the Ag^+^ liberation from the AgNPs plays a predominant role. When the synergy is stronger for the AgNO_3_ than for the AgNPs, the nanoparticle structure may limit this liberation, or the corona somehow prevents a higher level of synergy. Finally, when the AgNPs are more active in producing synergy than the silver ions, the AgNPs themselves are involved in the synergy and not only the silver ions generated from them. Since we found that the lack of synergy of some antibiotics with all the AgNPs types correlates with the result obtained for AgNO_3_, except for tetracycline against *E. coli*, we would expect that if the synergy depends on the liberation of Ag^+^ from the AgNPs, as some authors have claimed [136], the lack of synergy of AgNO_3_ with an antibiotic will result in the same for any AgNPs with that antibiotic. For the cases when AgNO_3_ does not raise synergy but the AgNPs do, this would mean that another mechanism related to the AgNPs themselves is involved in the synergy, as could be happening with tetracycline in our study.

Many publications have reported the synergistic action of various AgNPs with several antibiotics against *E. coli*, some of which have been reviewed by several authors [10,20,42,129]. In particular, for the antibiotics tested herein, considering studies performed using the chequerboard method through the determination of the FICI, the negative results for synergy of any of the AgNPs reported here with ampicillin agreed with several other analyses [106,138,139,140,141], as well as with our previous studies using AgNPs of different origins [55,56]; however, others have found synergy [128,138,142]. In a report by Mohammed et al. [128], the synergy of AgNPs with ampicillin was observed against 30 out of 35 extended-spectrum beta-lactamases (ESBL)-producing *E. coli* isolates. Hwang et al. [142] found synergy of AgNPs with chloramphenicol, in agreement with our results, but Vazquez-Muñoz et al. [139] reported a lack of it. Very few studies have evaluated the synergy of AgNPs with colistin. In agreement with our results, Alotaibi et al. [106] and Smékalová et al. [143], based on FICI values, found synergy of one AgNP type out of two, and Panáček et al. [110] and Dove et al. [103] found synergy via MIC comparison. Other studies using the ZOI method claimed synergy even though the diameter of the inhibition zone produced by the combination of the antibiotic and the AgNPs was smaller or the same as the sum of those produced by each antibacterial agent independently. The reason for this is probably that the inhibition produced by the AgNPs alone was not considered for the analysis of the results, leading to their conclusions [127,144,145], as it is frequent in studies performed using the ZOI approach [43]. It is worth mentioning that Wali et al. [8] studied the synergy of AgNPs and colistin through in vivo experiments using a burn wound-infected rat model, with promising results against *P. aeruginosa* and *K. pneumoniae*. The results published for ciprofloxacin showed synergy with AgNPs by Panáček et al. [146], as for two of the AgNPs obtained in the present work, and 91.4% of the ESBL-producing isolates of *E. coli* that were analysed by Mohammed et al. [128]; however, results were negative in studies by Alotaibi et al. [106], Hochvaldová et al. [147], Hassan et al. [148] and Rastogi et al. [149], as well as for another two of the AgNPs reported in this study. Ankudze and Neglo [150] even claimed antagonist behaviour between their AgNPs and this antibiotic. As in our study, a lack of synergy with ceftazidime was reported by Haji et al. [151] and Hochvaldová et al. [147], although a synergistic effect was claimed by Panácek et al. [146] and against 32 out of 35 ESBL-producing *E. coli* isolates obtained by Mohammed et al. [128]. Most studies using ZOI or MIC fold methods did not detect synergy with this antibiotic [10,20,42,129]. The only report we found analysing the putative synergy of AgNPs with erythromycin by the chequerboard method concluded with a negative result [148], as we found with half of our AgNPs. We did not find in the literature any report assessing the synergy of AgNPs with ertapenem besides our previous study [55], which has produced negative results. However, other researchers evaluated the synergy of AgNPs with other carbapenem antibiotics. For instance, meropenem has shown no synergy in the article by Panácek et al. [146], but another report using the MIC folds method claimed a synergistic effect of other AgNPs with this antibiotic [110]. Moreover, AgNPs in combination with imipenem [151] or biapenem [139] did not generate synergy. Finally, using the ZOI method, Ghosh et al. [152] found negative results for synergy with faropenem, and Dove et al. [103] found negative results for synergy with imipenem and doripenem using the MIC fold method. For kanamycin, some papers reported synergy [55,106,139,142,153], as in our current study, while others showed negative results [141,154]. As far as we know, for nalidixic acid, synergy has only been studied by Ghosh et al. [152], who used the ZOI method and claimed synergistic behaviour, in disagreement with the results found for the four types of AgNPs reported in the current article. Moreover, our previous studies with this antibiotic using the chequerboard method also showed no synergy for another twelve types of AgNPs [55,56]. Murei et al. [138] described AgNPs as synergic with penicillin, but another report showed no synergistic effect with penicillin G [143]. A study found synergy of AgNPs with rifampicin using the chequerboard method [106], which agrees with our current results. Nevertheless, most studies with this antibiotic used the ZOI approach, with positive results in most cases [155,156,157,158], but in one case, a negative result was reported [152]. Several studies supported synergy for AgNPs in combination with streptomycin using the chequerboard method [55,56,149,153,154], as in this study. For tetracycline, research based on the FICI evaluation reported an AgNP type synergising with the antibiotic, but this was not the case for the AgNPs that were evaluated by Wypij et al. [141]. In our previous study using the same method, all the evaluated AgNPs showed synergy with this antibiotic against *E. coli* [55]. One report using the MIC folds method [103] claimed no synergy, and Ankudze and Neglo [150] reported an antagonist behaviour. We found only two studies using the chequerboard method to assess the putative synergy of AgNPs with tigecycline. Neither Al-Otibi et al. [159] nor Wan et al. [137] found support for synergy, as in the study presented here. Murei et al. [138] evaluated two types of AgNPs for synergy with vancomycin, one chemically synthesised and another biogenic, obtaining a positive and a negative result, respectively. In another study, Alotaibi et al. [106] reported synergy, but in another one, the AgNPs used did not show synergy [148]. Finally, other studies using the analysis of ZOI, MIC folds, and colony counting have also shown positive or negative results for synergy between the antibiotics used in this work and various AgNP types [10,20,42,129].

A study on the synergy of the AgNPs and AgNO_3_ against the Gram-positive bacterium *S. aureus* (Table 4) were positive when combining AgNPs with ampicillin (FICI = 0.375–0.500 and MF = 4–2) and penicillin G, (FICI = 0.188–0.313 and MF = 8–4), both being members of the beta-lactam family and the penicillin group [160], presenting both related structures and mechanisms of action, even though different levels of the FICI were found, suggesting higher synergy with penicillin G than with ampicillin. Synergy was also detected with colistin, with low values of FICI (FICI = 0.062–0.094 and MF = 32), with kanamycin (FICI = 0.046–0.094 and MF = 32–64), and with streptomycin (FICI = 0.031–0.094 and MF = 16–64). With ciprofloxacin, only two AgNPs (LY-E-AgNPs and LY-S-AgNPs) reached FICI = 0.500, indicating synergy. The AgNPs gave lower FICI values than AgNO_3_ for colistin and kanamycin. Still, it was the contrary for ampicillin, penicillin G and streptomycin, while ciprofloxacin did not work synergistically with ionic silver but did with two types of nanosilver. It remains to elucidate whether these differences have a meaning concerning the mode of action of the antibiotics, the AgNPs, or the AgNO_3_ in combination.

The results showed that a larger spectrum of antibiotics increased their activity in the presence of the AgNPs against *E. coli* than against *S. aureus*. Moreover, three antibiotics did not show synergy with the silver ions or any of the AgNPs against both bacteria, ceftazidime, nalidixic acid, and tigecycline, and only three synergised with the AgNPs or AgNO_3_ against both types of tested bacteria: colistin, kanamycin, and streptomycin, being the synergy observed specific against *E. coli* in the cases of chloramphenicol, rifampicin, tetracycline, and vancomycin, and for *S. aureus* with ampicillin and penicillin G. In some cases, this specificity was only for some of the AgNPs, as in the case of ciprofloxacin against *S. aureus* (Ly-E-AgNPs and LY-S-AgNPs) and for erythromycin and ertapenem, with two AgNPs (LY-SCl-AgNPs and LY-S-AgNPs) or one (LY-S-AgNPs), respectively, against *E. coli*.

Several authors have studied combinations of various AgNPs with the same antibiotics we evaluated herein using *S. aureus* as a test species. Thus, the synergy of AgNPs with ampicillin found in our study agrees with the observations by Wang et al. and Hwang et al. [136,142]; however, Lopez-Carrizales et al. [140] reported a negative result, while Wypij et al. [141] found that one type of AgNP produced synergy but another not. Other studies not using the chequerboard method reported positive and negative results for the synergy of AgNPs with ampicillin against *S. aureus*. We did not find a synergy in combinations of our AgNPs and chloramphenicol, as found by Vazquez-Muñoz et al. [139] and Hwang et al. [142], but the study by Hadi et al. [161] using the ZOI method suggested synergy between their AgNPs and this antibiotic. The AgNO_3_ and the AgNPs synthesised in our work synergised with colistin against *S. aureus*. Colistin is a polymyxin antibiotic that acts by binding to components of the outer membrane, which are only present in Gram-negative bacteria [162], so their use against Gram-positive bacteria is discouraged because of their lack of efficacy. Perhaps this could be why, as far as we know, there is no study testing AgNPs’ synergy with colistin against *S. aureus*. Nevertheless, some authors found that using this antibiotic against biofilms of some strains of *S. aureus* can liberate live cells by affecting the biofilm–matrix structure [163] via an unknown mechanism, facilitating their killing with other anti-S. *aureus* drugs. Since we detected that silver can produce a synergistic effect with this antibiotic against planktonic cells of this Gram-positive bacterium, even with a lower FICI than for *E. coli*, and a reduction in the colistin MIC from 512 to 16 µg/mL (MF = 32) in the synergy conditions, we concluded that this antibiotic could be used to fight against *S. aureus*, expanding the spectrum of action of colistin, and perhaps even combining effects on planktonic cells and biofilms, which may be worth testing in the future. Only two of our AgNPs showed synergy with ciprofloxacin, but with FICI values on the limit of positive synergy. No synergy was found by Hassan et al. [148], Rastogi et al. [149], and in our previous works [55,56] but, on the contrary, AgNPs described by Wang et al. and Ankudze and Neglo [136,150] showed synergistic behaviour, as well as those analysed by Panáček et al. [146] in the MIC changes and by Naqvi et al. [164] based on the ZOI method. Aabed and Mohammed [78] evaluated the putative synergy of two different AgNPs with ciprofloxacin, finding that they have called a “slight synergistic” effect on the ZOIs for one of them and an antagonistic one for the other. Against *S. aureus*, ceftazidime-AgNPs (or AgNO_3_) combinations showed high FICI values, so neither AgNO_3_ nor AgNPs had any synergy with this antibiotic. We did not find any study that uses the chequerboard method to assess AgNPs’ synergy with ceftazidime, but a study using the ZOI method suggested synergy [156]. Erythromycin did not work synergistically with any of the silver-containing materials used in our study, in agreement with the negative results reported by Hassan et al. [148], but not with the positive ones obtained by Mishra et al. [131], both using the chequerboard assay. Neither silver ions nor AgNPs were able to synergise with ertapenem in our study, and, up to now, no other article has reported a study with this antibiotic. However, some studies tested nanosilver materials for synergy with antibiotics of the same family—for instance, the synergy of AgNPs with biapenem, analysed by Vazquez-Muñoz et al. [139] using the calculated FICI values, with imipenem by Naqvi et al. [164], and with faropenem by Ghosh et al. [152], both using the ZOI method, all reported no synergy. As in our work, Wang et al. [136], Vazquez-Muñoz et al. [139], Hwang et al. [142], and against one of the two *S. aureus* strains tested by Trzcińska-Wencel et al. [132], reported the synergy of AgNPs with kanamycin, while Wypij et al. [141] and Barapatre et al. [154] observed synergy with one but not with another type of AgNPs in both articles. Studies of the synergy of AgNPs with nalidixic acid are scarce, and we found only one against *S. aureus,* in which Ghosh et al. [152] reported only a slight increase in the ZOI between those produced by the antibiotic in the presence and absence of the AgNPs, with a negative result found for all the AgNPs reported here. The synergy of two types of AgNPs with penicillin G, which was studied using the chequerboard method by Smékalová et al. [143], was negative, but our AgNPs showed strong synergy. In other studies using MIC or ZOI methods, positive or negative results for synergy were reported—for instance, in [110] and [158], respectively. As far as we know, evaluating the synergy of AgNPs with rifampicin against *S. aureus* was only reported by Patra et al. [157] using the ZOI method, claiming a positive synergy that does not agree with the results obtained with any of the AgNPs presented in this article. The synergy with streptomycin found in our work was also reported by Rastogi et al. [149] for two types of AgNPs and by Barapatre et al. [154] for one out of two AgNP types. Moreover, Trzcińska-Wencel et al. [132] found negative results against two *S. aureus* strains. These three articles extracted their conclusions from the corresponding FICI values. Trzcińska-Wencel et al. [132] detected no synergy when using a combination of AgNPs and tetracycline against two strains of *S. aureus* for the four AgNP types described in this article. However, Wang et al. [136] reported a synergistic behaviour both for Ag^+^ and AgNPs and Wypij et al. [141], wherein one of the two AgNPs types they have evaluated showed synergy with this antibiotic. Several studies using the ZOI or MIC methods reported negative or positive results for synergy between AgNPs and this antibiotic—for instance, in [78] and [165], respectively. Moreover, Ipe et al. [166] obtained positive or negative results for the same AgNPs against two strains of *S. aureus*. A study using the chequerboard and the ZOI method found no synergy between AgNPs and tigecycline against *S. aureus*, as in the current study. Further studies could establish if this lack of synergy occurs for any AgNPs or depends, as for other antibiotics, on the AgNPs’ characteristics. In synergy studies of AgNPs with vancomycin, Hassan et al. [148] found a negative result both using the chequerboard, as in our case, and ZOI methods. Mishra et al. [131] described strong synergy in the low FICI values obtained. Other authors, using ZOI or MIC analyses, claimed a synergy, as in [167], or no synergy, as in [125]. Using the synergy experimental results described in this report and the analysis of other researchers’ results, we can deduce that AgNP-antibiotic synergy depends on the AgNPs, the species, and even the strain tested. The different methods used for in vitro synergy assessment by various authors are a handicap for comparative studies. If we eventually want to use the synergistic approach for practical use in fighting infections, it is mandatory to establish standard, universal and quantitative analytic methods. Given this, the results of different studies could be easily compared to obtain relevant information about which AgNPs synergise with which antibiotic and against which species of bacteria. We will have, for instance, to discard those AgNPs whose synergy behaviour with a specific antibiotic is positive or negative depending on the strain evaluated of a bacterial species. Only those with a strain-independent synergistic behaviour will be useful in practice, since otherwise, the output of an infection treatment would be uncertain because we would not know whether synergy would work for the specific strain involved. Moreover, AgNPs producing synergy against several species will sometimes be even better from a practical point of view. It would also be interesting to determine the spectrum of action of each AgNP-antibiotic combination.

### 3.8. ROS Production and Bacteria Killing by the AgNPs

One of the most controversial aspects in the study of AgNPs is the mechanism by which they produce their antibacterial effect. Several authors revised the literature on this subject in the last few years [39,168,169,170,171]. However, there is still no consensus on this matter because it is difficult to analyse. Several methods sometimes generate contradictory results, suggesting different mechanisms acting individually or in combination to produce bacterial death. Moreover, several of these mechanisms can be closely related and may feed back on each other, making it difficult to determine the principal or original cause of the antibacterial effect [169,172].

Among the claimed mechanisms, some authors think that the principal causes of the AgNPs’ antibacterial activity are the instability of the plasma membrane and, consequently, the increase in cell permeability [173,174,175] or the interaction with bacterial DNA [175,176,177,178]. However, most authors point out that the induction of ROS in the presence of the AgNPs is responsible for this activity [35,39,171,179,180,181,182]. Concerning this topic, we started to analyse the relationship between the ROS production by *E. coli* and *S. aureus* during their treatment with several types of biogenic AgNPs and their MICs [56], and, more recently, between ROS production and bacterial cell survival after treatment with several other types of biogenic AgNPs [55].

**Figure 11:**
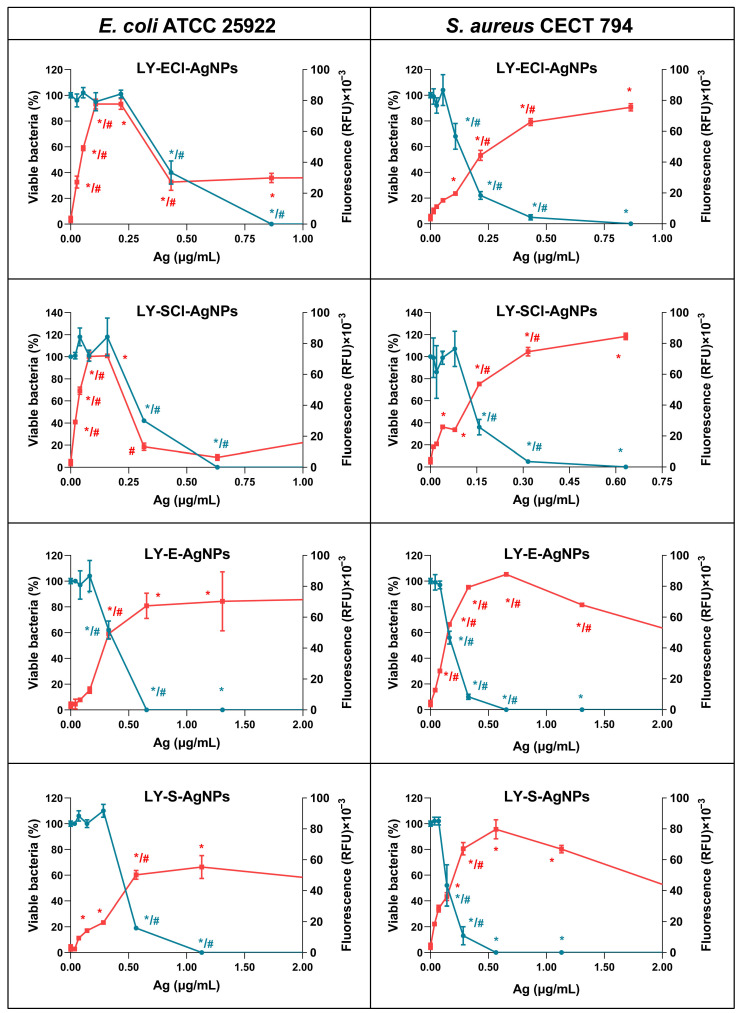
ROS production and viable cells after treatment with AgNPs. Fluorescence is shown in red, and the % of viable bacteria is shown in blue. Statistically significant differences of each value with the untreated control (*) and the immediately lower AgNPs’ concentration value (#) are indicated.

Our current study showed that increasing the AgNPs concentration resulted in a significant and dose-dependent increase in ROS production (Figure 11). This correlation agrees with the widespread idea that AgNPs produce a ROS accumulation directly or indirectly in bacterial cultures [183], a phenomenon that has also been described in eukaryotic animal cells [184,185]. However, above some AgNPs concentrations, the ROS accumulation decreased, as can be seen for AgNPs from chlorine-containing media when testing *E. coli* and AgNPs from non-chlorine-containing media against *S. aureus* (Figure 11). Other authors described similar results by using different types of AgNPs when testing against *Pseudomonas stutzeri* [183] or *E. coli* [186]. These authors proposed that this observed drop in fluorescence (interpreted as a reduction of ROS production) is due to a methodological problem, as the high concentrations of AgNPs could mask the fluorescence of DCFH-DA used in the ROS detection experiments. However, the fact that the drop in fluorescence occurs at different concentrations of the same type of AgNPs for each control bacterium and that it always occurs at the concentrations at which a significant bactericidal effect is observed (Figure 11) indicate that the reason is rather that the cells die more effectively at these concentrations and do not produce sufficient ROS accumulation to reach values similar to the maximum values obtained at lower and not so efficient for killing concentrations. In our previous study, we also observed this effect [55].

The ranges of maximum ROS accumulation were very similar in both control bacteria for the various AgNPs. Wu et al. found that the effect of AgNPs on the level of ROS production was different depending on the evaluated bacterium [35]. These researchers found that the ROS increase was lower in *S. aureus* than in *E. coli*, and their AgNPs did not produce as much ROS as the AgNO_3_ itself. This suggests that the ROS production is dependent on the AgNPs tested and even the strain of the species used. Our previous studies [55,56] yielded similar results. However, the AgNPs produced in the current work did not show a relevant difference in the maximum level of ROS accumulation, and the slight difference found was of higher accumulation in *S. aureus* than in *E. coli*. Since we used the same strains and protocols in our previous work and the current one, we can conclude that the characteristics of the new AgNPs are responsible for this result.

The level of ROS accumulation by one or the other tested species did not present an apparent correlation with any specific physicochemical characteristic of the different AgNPs, which does not confirm what other authors have claimed. For example, several authors consider that the smaller the size of the nanoparticles, the higher the ROS production and, therefore, the higher their antibacterial activity [35,170,187] or cytotoxic effect [188]. As any of the measured physicochemical characteristics of our AgNPs correlated with the differences in ROS production or antibacterial activity, we should consider the possibility of other parameters, such as the specific composition of the AgNPs’ coronas, being more relevant.

The AgNPs concentration needed for maximum ROS production depended on the AgNPs type and the control bacteria used (Figure 11). Against *E. coli*, AgNPs from chlorine-containing media required lower concentrations to induce maximum ROS production (0.216 and 0.158 µg/mL) than their counterparts from non-chlorine-containing media (0.65 and 1.13 µg/mL), indicating a higher ROS-generating efficiency of the first ones that may be related to the relative antibacterial activities observed for these groups of AgNPs. However, against *S. aureus,* concentrations producing maximum ROS accumulation were similar (0.560–0.650 µg/mL) for one of the AgNPs from broth with chloride and both of the AgNPs from broths without that element. Only the LY-ECl-AgNPs presented a differentiated and higher value (0.860 µg/mL). Thus, the same AgNPs had different comparative efficacies in promoting ROS accumulation depending on the control bacteria used. Furthermore, the concentrations of AgNPs that produced a complete bacterial killing (0% viable cells detected) did not present any more remarkable differences between AgNPs.

When studying the relationship between ROS production and cell viability (Figure 11), the decrease in cell viability correlated with the increase in ROS accumulation for both evaluated bacteria when treated with the AgNPs without Cl^−^, like the results obtained in a previous report with AgNPs from *Parachlorella* sp. cultures without Cl^−^ [55]. However, the AgNPs produced with broths with Cl^−^ (LY-ECl-AgNPs and LY-SCl-AgNPs) revealed a different behaviour against *S. aureus* or *E. coli*. For the former, ROS production correlated with the decrease in CFUs, as with the AgNPs without chloride, while against *E. coli*, the drop in viability was only achieved when the AgNPs concentration produced the maximum ROS accumulation. So, only the maximum ROS accumulation killed the cells, while lower AgNPs concentrations did not generate any evident decrease in cells’ viability. Besides these two differentiated behaviours of particular AgNPs, our previous work revealed the existence of another different mechanism against *E. coli* and *S. aureus* for AgNPs produced with *Parachlorella* sp. cultures in a medium containing Cl^−^ [55]. In this mechanism, the maximum bacterial killing appeared at lower concentrations than those that resulted in maximum ROS accumulation. All these data, taken together, support that the ROS accumulation may or may not be the unique mechanism involved in the bacterial killing by AgNPs, depending on the AgNPs and the bacteria. For AgNPs described by other authors, ROS production played a fundamental role in the antibacterial activity of some of them [39,171,179,181,182]. Nevertheless, other researchers argued a mechanism involving cell envelope permeability changes [173,174,180] or other mechanisms [41].

Our results could explain why there are so many discrepancies in the mechanism of action of AgNPs claimed by different authors. Given this, the AgNPs and the bacteria determine whether one mechanism or another is principal in the antibacterial activity of AgNPs. Therefore, it is of great interest to test AgNPs with different characteristics and against different control bacteria to establish the importance of each mechanism in the behaviour of each AgNP type and to find the reasons for such differences to design better and more efficient antibacterial agents.

## 4. Conclusions

In this report, we describe the extracellular synthesis of four types of AgNPs prepared from culture broths of a bacterial isolate belonging to an undescribed species of the *Lysinibacillus* genus; their physicochemical characterisation concerning elemental composition, shape, core and hydrodynamic sizes, Z-potential, crystallinity, and corona composition, as well as their antibacterial activity against three Gram-positive and three Gram-negative bacteria; their effect on the antibacterial activity of 15 classical antibiotics against *E. coli* and *S. aureus*; and their impact on ROS production and the correlation of this with their bacteria-killing capabilities on *E. coli* and *S. aureus* cells.

We used four *Lysinibacillus* sp. culture broths corresponding to media with or without NaCl and from the exponential and stationary growth phases to produce different types of AgNPs, the composition of which was mainly silver with small amounts of chlorine that depended on the presence or absence of NaCl in the broths. Furthermore, all the AgNPs presented a quasi-spherical shape and cores in a small size range, with negative Z-potentials, and contained metallic silver crystals, and some of them contained AgCl. In this work, we observed that the broths used for the AgNPs influence the physicochemical and other characteristics, such as their antibacterial activity.

An evaluation of the AgNPs’ antibacterial activity showed their capability to inhibit the planktonic growth and the biofilm formation of three Gram-negative and three Gram-positive bacteria, measured by their MIC, MBC, IC_50_, and ICb_50_ parameters. These indicated an excellent activity, with some differences for the four tested AgNPs types. Moreover, the study of the antibacterial activity of combinations of AgNPs and 15 classical antibiotics allowed for calculating the FICI values, from which a synergistic effect of the AgNPs on the activity of the antibiotics chloramphenicol, colistin, kanamycin, rifampicin, streptomycin, and tetracycline against *E. coli* and ampicillin, colistin, kanamycin, penicillin G, and streptomycin against *S. aureus* derived. Among the most interesting results obtained, the activity of streptomycin against a resistant strain of *S. epidermidis* improved, and colistin reduced its MIC 32 times against *S. aureus*, a species usually not affected by this antibiotic.

The analysis of the ROS accumulation in *E. coli* and *S. aureus*, when treated with AgNPs, showed its correlation with the killing capability of the AgNPs for all of them against *S. aureus,* but only for some against *E. coli*. Two AgNPs generated ROS accumulation in *E coli* that did not produce any bacterial cell killing until the ROS accumulation reached the maximum, indicating that other mechanisms besides ROS can intervene in the antibacterial activity of these AgNPs.

## Figures and Tables

**Table 1:** Naming of the AgNPs according to the broths used in their synthesis.

Microorganism	Culture Medium	Culture Phase	Name of AgNPs
*Lysinibacillus* sp.	Nutritive medium with NaCl	Exponential	LY-ECl-AgNPs
		Stationary	LY-SCl-AgNPs
	Nutritive medium without NaCl	Exponential	LY-E-AgNPs
		Stationary	LY-S-AgNPs

**Table 2:** Physicochemical parameters of AgNPs (mean ± standard deviation).

AgNPs	Potential-Z (mV)	Diameter (DLS) (nm)	PDI (DLS)	Diameter (TEM) (nm)	PDI (TEM)
LY-ECl-AgNPs	−20.6 ± 1.6	48.5 ± 0.4	0.45	9.9 ± 5.5	0.31
LY-SCl-AgNPs	−21.6 ± 5.4	52.7 ± 0.5	0.51	14.7 ± 8.3	0.32
LY-E-AgNPs	−25.1 ± 3.5	80.2 ± 3.6	0.43	7.5 ± 5.8	0.50
LY-S-AgNPs	−8.8 ± 1.4	63.3 ± 1.7	0.45	9.6 ± 5.1	0.28

**Table 3:** Antibacterial parameters of AgNPs/AgNO_3_ against the test bacteria (µg/mL).

Test Bacteria	AgNPs/AgNO_3_	MIC	MBC	IC_50_	ICb_50_
*E. coli* ATCC 25922	Streptomycin	16.00	16.00	5.40 ± 1.06	5.83 ± 1.52
	AgNO_3_	0.53	0.53	0.15 ± 0.01	0.33 ± 0.03
	LY-ECl-AgNPs	0.86	1.73	0.43 ± 0.06 *	0.62 ± 0.04 */#
	LY-SCl-AgNPs	0.63	1.26	0.25 ± 0.02 *	0.34 ± 0.02 *
	LY-E-AgNPs	0.65	0.65	0.43 ± 0.07	0.35 ± 0.02 #
	LY-S-AgNPs	1.13	1.13	0.55 ± 0.21	0.56 ± 0.20
*K. pneumoniae* ATCC 29665	Streptomycin	4.00	4.00	2.59 ± 0.18	2.29 ± 0.38
	AgNO_3_	0.53	0.53	0.27 ± 0.01	0.30 ± 0.07
	LY-ECl-AgNPs	0.86	3.46	0.49 ± 0.11	0.50 ± 0.02
	LY-SCl-AgNPs	1.31	2.61	0.41 ± 0.09	0.73 ± 0.15 #
	LY-E-AgNPs	1.31	2.61	0.45 ± 0.50	0.78 ± 0.20
	LY-S-AgNPs	2.26	2.26	0.61 ± 0.41	1.11 ± 0.04 #
*P. aeruginosa* CECT 108	Streptomycin	16.00	16.00	4.50 ± 0.27	6.43 ± 0.75
	AgNO_3_	0.27	0.53	0.03 ± 0.01	0.16 ± 0.02
	LY-ECl-AgNPs	0.43	1.73	0.13 ± 0.02 *	0.25 ± 0.02 *
	LY-SCl-AgNPs	0.32	0.63	0.08 ± 0.01 */#	0.18 ± 0.01 */#
	LY-E-AgNPs	1.31	1.31	0.16 ± 0.12	0.45 ± 0.21
	LY-S-AgNPs	0.57	0.57	0.36 ± 0.06 #	0.42 ± 0.01 #
*S. aureus* CECT 794	Streptomycin	32.00	32.00	4.15 ± 0.11	5.45 ± 0.92
	AgNO_3_	2.12	4.24	0.56 ± 0.02	1.28 ± 0.15
	LY-ECl-AgNPs	6.92	13.83	1.54 ± 0.05 *	1.76 ± 0.16 #
	LY-SCl-AgNPs	5.06	10.12	0.87 ± 0.26 */#	2.04 ± 0.36 #
	LY-E-AgNPs	5.23	10.46	1.65 ± 0.23 *	2.88 ± 0.18 */#
	LY-S-AgNPs	9.05	18.10	3.24 ± 0.26 */#	5.20 ± 0.27 */#
*S. epidermidis* ATCC 12228	Streptomycin	>256.00	>256.00	>256.00	>256.00
	AgNO_3_	1.06	4.24	0.27 ± 0.01	0.90 ± 0.35
	LY-ECl-AgNPs	1.73	6.92	0.48 ± 0.08 #	0.92 ± 0.21 #
	LY-SCl-AgNPs	1.26	5.06	0.42 ± 0.06 #	0.93 ± 0.20
	LY-E-AgNPs	2.61	10.46	1.46 ± 0.14 */#	1.71 ± 0.24 #
	LY-S-AgNPs	4.52	36.20	0.67 ± 0.08 */#	1.46 ± 0.71
*B. subtilis* 168	Streptomycin	64.00	>256.00	7.58 ± 0.82	14.09 ± 5.80
	AgNO_3_	1.06	1.06	0.27 ± 0.08	0.40 ± 0.13
	LY-ECl-AgNPs	1.73	1.73	0.95 ± 0.16 #	1.45 ± 0.21 *
	LY-SCl-AgNPs	2.61	5.06	0.75 ± 0.10	0.83 ± 0.14 */#
	LY-E-AgNPs	2.61	10.46	2.88 ± 0.58 #	1.19 ± 0.07 *
	LY-S-AgNPs	4.52	9.04	2.52 ± 0.91	2.65 ± 0.48 */#

MIC: minimal inhibitory concentration; MBC: minimal bactericidal concentration; IC_50_: 50% growth inhibitory concentration; ICb_50_: 50% biofilm formation inhibitory concentration; * indicates significant differences between AgNPs prepared from the same medium but different growth phase; # indicates significant differences between AgNPs from the same growth phase but from different media. *p*-value: <0.05 (*) or (#).

**Table 4:** Synergistic effect between AgNPs/AgNO_3_ and antibiotics (FICI and MF).

Antibiotic		AgNO_3_		LY-ECl-AgNPs		LY-SCl-AgNPs		LY-E-AgNPs		LY-S-AgNPs	
		*E.C.*	*S.A.*	*E.C.*	*S.A.*	*E.C.*	*S.A.*	*E.C.*	*S.A.*	*E.C.*	*S.A.*
Ap	FICI	2.000	0.250	0.625	0.500	2.000	0.375	1.000	0.375	2.000	0.375
	MF	1	8	2	2	1	4	2	4	1	4
Cc	FICI	0.375	0.750	0.313	2.000	0.188	2.000	0.250	2.000	0.375	1.000
	MF	4	4	4	1	8	1	8	1	4	2
Co	FICI	0.188	0.094	0.157	0.063	0.125	0.063	0.094	0.063	0.094	0.063
	MF	16	32	8	32	16	32	32	32	32	32
Cp	FICI	2.000	1.000	2.000	0.750	2.000	0.750	2.000	0.500	2.000	0.500
	MF	1	2	1	2	1	2	1	4	1	4
Cz	FICI	2.000	3.000	2.000	3.000	2.000	3.000	2.000	3.000	2.000	3.000
	MF	1	1	1	1	1	1	1	1	1	1
Em	FICI	1.000	2.000	0.750	0.750	0.500	0.750	0.750	2.000	0.500	2.000
	MF	2	1	2	2	4	2	4	1	4	1
Ep	FICI	0.750	0.750	2.000	2.000	1.000	2.000	1.000	2.000	0.500	2.000
	MF	2	2	1	1	2	1	2	1	4	1
Km	FICI	0.047	0.094	0.039	0.078	0.039	0.078	0.039	0.046	0.035	0.078
	MF	64	32	128	64	128	64	128	64	128	32
Nx	FICI	1.000	2.000	2.000	1.000	1.000	2.000	2.000	2.000	1.000	2.000
	MF	2	1	1	2	2	1	1	1	2	1
Pn	FICI	0.750	0.078	1.000	0.188	0.750	0.188	1.000	0.188	1.000	0.313
	MF	2	64	2	8	4	8	2	8	2	4
Rp	FICI	0.188	1.000	0.158	1.000	0.094	1.000	0.125	1.000	0.188	1.000
	MF	16	2	8	2	16	2	16	2	16	2
Sm	FICI	0.047	0.031	0.063	0.047	0.180	0.125	0.125	0.094	0.094	0.094
	MF	64	64	64	64	16	16	16	32	32	32
Tc	FICI	0.750	0.750	0.375	1.000	0.500	2.000	0.310	2.000	0.375	0.750
	MF	2	4	4	2	4	1	16	1	4	4
Tg	FICI	2.000	2.000	2.000	0.750	2.000	0.750	2.000	0.750	1.000	1.000
	MF	1	1	1	2	1	2	1	2	2	2
Vm	FICI	0.375	0.750	0.625	1.000	0.375	1.000	0.375	0.750	0.375	1.000
	MF	8	2	2	2	4	2	4	4	4	2

Test bacteria: *Escherichia coli* ATCC 25922 (*E.C.*) and *Staphylococcus aureus* CECT 794 (*S.A.*). FICI: fractional inhibitory concentration index; MF: modulation factor. Antibiotic abbreviations are indicated in Section 2.5.

## Data Availability

The original contributions presented in this study are included in the article/Supplementary Materials. Further inquiries can be directed to the corresponding authors.

## Supplementary Materials

The following supporting information can be downloaded at: https://www.mdpi.com/article/10.3390/biom15050731/s1, Figure S1: FICI and MF calculation; Figure S2: Intensity distribution curves of AgNPs’ hydrodynamic sizes; Table S1: Antibacterial activity of the AgNPs conserved in the dark at 4 °C; Table S2: Susceptibility of several *P. aeruginosa* strains to LY-S-AgNPs; Table S3: MICs (µg/mL) of the antibiotics alone or in combination with AgNO_3_/AgNPs.
