# Supplementary material for: Silver Nanoparticles (AgNPs) from Lysinibacillus sp. Culture Broths: Antibacterial Activity, Mechanism Insights, and Synergy with Classical Antibiotics
Source: Biomolecules. 2025 May 16;15(5):731. doi: 10.3390/biom15050731 (PMC12109054; doi:10.3390/biom15050731)
Supplement: Supplementary file 1 [file biomolecules-15-00731-s001.zip › biomolecules-3551922-supplementary.pdf]

$$FICI = \frac{MIC(A_B)}{MIC(A)} + \frac{MIC(B_A)}{MIC(B)}$$

$$MF = \frac{MIC(A)}{MIC(A_B)}$$

Figure S1. FICI and MF calculation. The MIC values correspond to A: antibiotic, B: silver-containing material, A_B to antibiotic in the presence of $AgNO_3$ or AgNPs, and B_A to silver-containing material in the presence of the antibiotic.

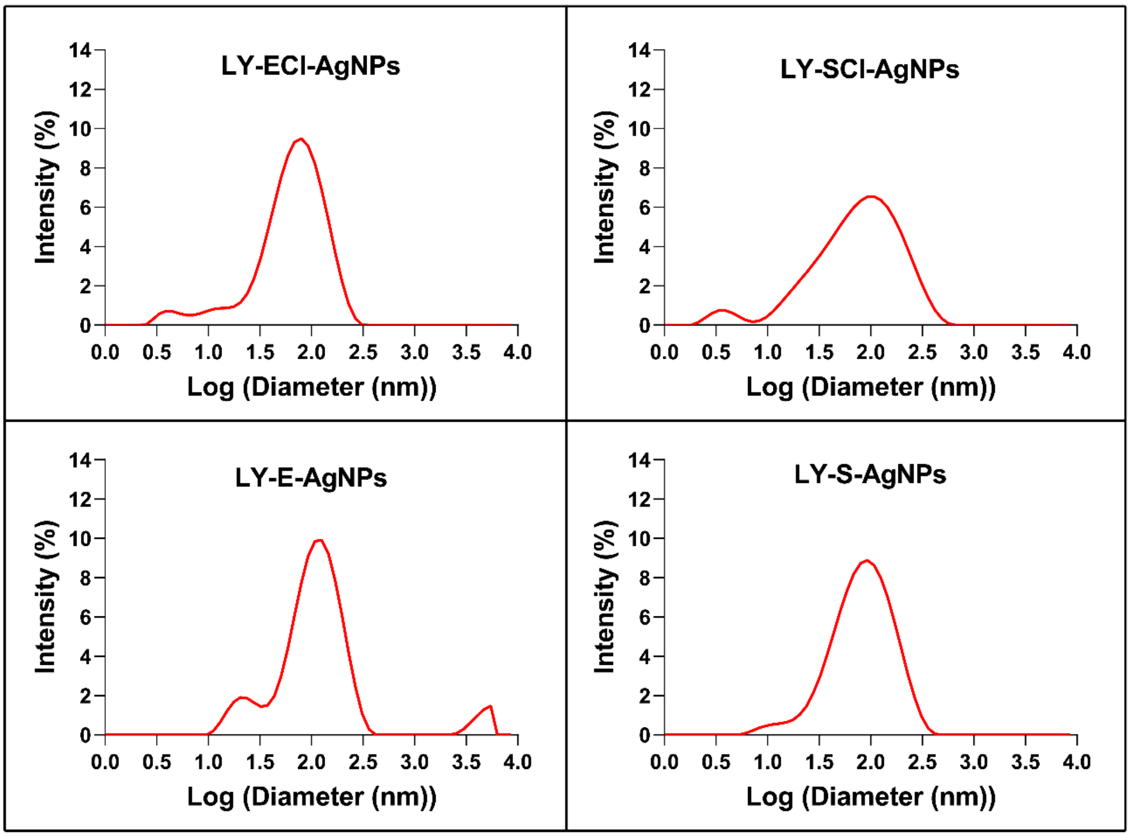

Figure S2. Intensity distribution curves of AgNPs' hydrodynamic sizes.

Table S1. Antibacterial activity of the AgNPs conserved in the dark at 4°C.

E. coli ATCC 25922	MIC (μg/mL) 0 Days	MIC (μg/mL) 30 Days	MIC (μg/mL) 730 Days
**LY-ECl-AgNPs**	0.86	0.86	0.43
**LY-SCl-AgNPs**	0.63	0.63	0.63
**LY-E-AgNPs**	0.65	0.65	0.65
**LY-S-AgNPs**	1.13	1.13	1.13

Table S2. Susceptibility of several *P. aeruginosa* strains to LY-S-AgNPs.

***P. aeruginosa* strain**	**MIC (μg/mL)**	**MBC (μg/mL)**	**IC_{50} (μg/mL)**	**ICb_{50} (μg/mL)**
**CECT 108**	0.56	0.56	0.30 ± 0.02	0.44 ± 0.09
**PA01**	0.56	0.56	0.32 ± 0.04	0.42 ± 0.08
**PA14**	0.56	0.56	0.28 ± 0.05	0.40 ± 0.04

Table S3. MICs (μg/ml) of the antibiotics alone or in combination with $AgNO_3$/AgNPs.

	***E. coli* ATCC 25922**						***S. aureus* CECT 794**					
**Ant.**	**Alone**	**$AgNO_3$**	**LY-ECl**	**LY-SCl**	**LY-E**	**LY-S**	**Alone**	**$AgNO_3$**	**LY-ECl**	**LY-SCl**	**LY-E**	**LY-S**
**Ap**	8.000	8.000	4.000	8.000	4.000	8.000	2.000	0.250	1.000	0.500	0.500	0.500
**Cc**	8.000	2.000	2.000	1.000	1.000	2.000	16.000	4.000	16.000	16.000	16.000	8.000
**Co**	2.000	0.125	0.250	0.125	0.063	0.063	512.000	16.000	16.000	16.000	16.000	16.000
**Cp**	0.030	0.030	0.030	0.030	0.030	0.030	0.500	0.250	0.250	0.250	0.125	0.125
**Cz**	2.000	2.000	2.000	2.000	2.000	2.000	64.000	64.000	64.000	64.000	64.000	64.000
**Em**	256.000	128.000	128.000	64.000	64.000	64.000	4.000	4.000	2.000	2.000	4.000	4.000
**Ep**	0.020	0.010	0.020	0.010	0.010	0.005	0.130	0.065	0.130	0.130	0.130	0.130
**Km**	6.250	0.098	0.049	0.049	0.049	0.049	6.250	0.195	0.098	0.098	0.098	0.195
**Nx**	4.000	2.000	4.000	2.000	4.000	2.000	64.000	64.000	32.000	64.000	64.000	64.000
**Pn**	32.000	16.000	16.000	8.000	16.000	16.000	0.500	0.008	0.063	0.063	0.063	0.125
**Rp**	8.000	0.500	1.000	0.500	0.500	0.500	0.020	0.010	0.010	0.010	0.010	0.010
**Sm**	16.000	0.250	0.250	1.000	1.000	0.500	32.000	0.500	0.500	2.000	1.000	1.000
**Tc**	1.000	0.500	0.250	0.250	0.063	0.250	0.250	0.063	0.125	0.250	0.250	0.063
**Tg**	0.500	0.500	0.500	0.500	0.500	0.250	1.000	1.000	0.500	0.500	0.500	0.500
**Vm**	256.000	32.000	128.000	64.000	64.000	64.000	1.000	0.500	0.500	0.500	0.250	0.500
